# Supplementary material for: Inverse Versus Normal Behavior of Interactions, Elucidated Based on the Dynamic Nature with QTAIM Dual-Functional Analysis
Source: Int J Mol Sci. 2023 Feb 1;24(3):2798. doi: 10.3390/ijms24032798 (PMC9917772; doi:10.3390/ijms24032798)
Supplement: Supplementary file 1 [file ijms-24-02798-s001.zip › ijms-2160471-supplementary.pdf]

Electronic Supplementary Information

Inverse versus Normal Behavior of Interactions, Elucidated Based on the Dynamic Nature with QTAIM Dual Functional Analysis

Waro Nakanishi,* Satoko Hayashi,* Ryosuke Imanaka, Taro Nishide, Eiichiro Tanaka and Hikaru Matsuoka

Faculty of Systems Engineering, Wakayama University, 930 Sakaedani, Wakayama 640-8510, Japan. E-mail: nakanisi@sys.wakayama-u.ac.jp (W.N.); hayashi3@sys.wakayama-u.ac.jp (S.H.)

Table of Contents

	Pages
Additional tables (**Tables S1–S12**)	S2–S16
Additional figures (**Figures S1–S6**)	S17–S19
Computation information and geometries of compounds	S20–S30
Appendix	S31–S36

Table S1. The interaction distances r_o(X, Y) in **1**–**36** optimized with MP2/BSS-A (aug-cc-pVTZ), MP2/BSS-B (6-311++G(3df,3pd)), and MP2/BSS-C (Sapporo-TZPsp).

Species (X-∗-Y) (No: symmetry)	$r_o(X, Y)_{BSS-A}$ (Å)	$r_o(X, Y)_{BSS-B}$ (Å)	$r_o(X, Y)_{BSS-C}$ (Å)	$\Delta r_o(X, Y)_{BSS-B}$ (Å)	$\Delta r_o(X, Y)_{BSS-C}$ (Å)
He-∗-HF (**1**: $C_{\infty v}$)	2.2738	2.2454	2.3743	-0.0283	0.1005
Ne-∗-HF (**2**: $C_{\infty v}$)	2.2783	2.1975	2.3724	-0.0807	0.0942
Ar-∗-HF (**3**: $C_{\infty v}$)	2.5109	2.5142	2.5748	0.0033	0.0639
Kr-∗-HF (**4**: $C_{\infty v}$)	2.5325	2.6423	2.5807	0.1098	0.0482
NN-∗-HF (**5**: $C_{\infty v}$)	2.0546	2.0293	2.0557	-0.0253	0.0011
HF-∗-HF (**6**: C_s)	1.8269	1.8196	1.8323	-0.0073	0.0055
HCN-∗-HF (**7**: $C_{\infty v}$)	1.8350	1.8238	1.8320	-0.0111	-0.0029
H_2O-∗-HOH (**8**: C_s)	1.9454	1.9427	1.9506	-0.0027	0.0051
Me_2O-∗-HOH (**9**: C_s)	1.8603	1.8636	1.8670	0.0033	0.0067
Me_2O-∗-Cl_2 (**10**: C_s)	2.5754	2.5545	2.6165	-0.0209	0.0411
Me_2O-∗-Br_2 (**11**: C_s)	2.5513	2.6003	2.5901	0.0490	0.0387
Me_2S-∗-Cl_2 (**12**: C_s)	2.5841	2.6378	2.6339	0.0537	0.0498
Me_2S-∗-Br_2 (**13**: C_s)	2.6555	2.6998	2.6735	0.0443	0.0181
Me_2Se-∗-Cl_2 (**14**: C_s)	2.5443	2.5693	2.5682	0.0250	0.0240
Me_2Se-∗-Br_2 (**15**: C_s)	2.6947	2.7433	2.7150	0.0486	0.0203
$[Cl\text{-}*\text{-}Cl_2]^-$ (**16**: $D_{\infty h}$)	2.3000	2.2956	2.2976	-0.0044	-0.0024
$[Br\text{-}*\text{-}Br_2]^-$ (**17**: $D_{\infty h}$)	2.5515	2.5594	2.5511	0.0079	-0.0004
$[Cl\text{-}*\text{-}BrCl]^-$ (**18**: $D_{\infty h}$)	2.4444	2.4061	2.4048	-0.0383	-0.0396
$[Br\text{-}*\text{-}ClBr]^-$ (**27**: $D_{\infty h}$)	2.4054	2.4464	2.4432	0.0410	0.0378
Me_2ClS-∗-Cl (**19**: C_2)	2.2751	2.2639	2.2691	-0.0112	-0.0059
Me_2BrS-∗-Br (**20**: C_{2v})	2.4426	2.4477	2.4411	0.0052	-0.0014
Me_2ClSe-∗-Cl (**22**: C_2)	2.3569	2.3560	2.3572	-0.0009	0.0003
Me_2BrSe-∗-Br (**23**: C_2)	2.5179	2.5314	2.5216	0.0135	0.0037
Me_2S^+-∗-Cl (**24**: C_s)	1.9950	1.9784	1.9862	-0.0166	-0.0088
Me_2S^+-∗-Br (**25**: C_s)	2.1521	2.1501	2.1473	-0.0020	-0.0048
Me_2Se^+-∗-Cl (**26**: C_s)	2.1170	2.1129	2.1111	-0.0041	-0.0059
Me_2Se^+-∗-Br (**27**: C_s)	2.2683	2.2757	2.2656	0.0073	-0.0028
Cl-∗-Cl (**28**: $D_{\infty h}$)	1.9987	1.9845	1.9917	-0.0142	-0.0070
Br-∗-Br (**29**: $D_{\infty h}$)	2.2787	2.2690	2.2756	-0.0097	-0.0031
CH_3-∗-Cl (**30**: C_{3v})	1.7805	1.7713	1.7780	-0.0091	-0.0025
CH_3-∗-Br (**31**: C_{3v})	1.9254	1.9294	1.9242	0.0040	-0.0012
CH_3-∗-CH_3 (**32**: S_6)	1.5242	1.5236	1.5227	-0.0006	-0.0016
CH_2-∗-CH_2 (**33**: D_{2h})	1.3332	1.3321	1.3318	-0.0011	-0.0014
CH-∗-CH (**34**: $D_{\infty h}$)	1.2122	1.2107	1.2121	-0.0014	0.0000
CH_3-∗-H (**35**: T_d)	1.0862	1.0854	1.0858	-0.0008	-0.0003
H-∗-H (**36**: $D_{\infty h}$)	0.7374	0.7366	0.7383	-0.0008	0.0009

[a] $\Delta r_o(X, Y)_{BSS-B} = r_o(X, Y)_{BSS-B} - r_o(X, Y)_{BSS-A}$. [b] $\Delta r_o(X, Y)_{BSS-C} = r_o(X, Y)_{BSS-C} - r_o(X, Y)_{BSS-A}$.

Table S2. The interaction distances r_o(X, Y) in **37–61**, optimized with MP2/BSS-A' (aug-cc-pVTZ + Sapporo-TZPsp), MP2/BSS-B'(6-311++G(3df,3pd) + Sapporo-TZPsp), and MP2/BSS-C (Sapporo-TZPsp).

Species (X-∗-Y) (No: symmetry)	$r_o(X, Y)_{BSS-A'}$ (Å)	$r_o(X, Y)_{BSS-B'}$ (Å)	$r_o(X, Y)_{BSS-C}$ (Å)	$\Delta r_o(X, Y)_{BSS-B'}$ (Å)	$\Delta r_o(X, Y)_{BSS-C}$ (Å)
Xe-∗-HF (**37**: $C_{\infty v}$)	2.5852	2.7335	2.7654	0.1482	0.1802
Me_2O-∗-I_2 (**38**: C_s)	2.6579	2.7219	2.7524	0.0641	0.0945
Me_2S-∗-I_2 (**39**: C_s)	2.8994	2.9524	2.9439	0.0530	0.0445
Me_2Se-∗-I_2 (**40**: C_s)	2.9628	3.0184	3.0000	0.0556	0.0372
Me_2Te-∗-F_2 (**41**: C_s)	2.1401	2.1617	2.1581	0.0216	0.0180
Me_2Te-∗-Cl_2 (**42**: C_s)	2.5620	2.5811	2.5768	0.0192	0.0148
Me_2Te-∗-Br_2 (**43**: C_s)	2.7446	2.7718	2.7634	0.0272	0.0188
Me_2Te-∗-I_2 (**44**: C_s)	3.0748	3.0841	3.0830	0.0092	0.0082
$[I$-∗-$I_2]^-$ (**45**: $D_{\infty h}$)	2.9331	2.9331	2.9331	0.0000	0.0000
$[F$-∗-$IF]^-$ (**46**: $D_{\infty h}$)	2.0715	2.0797	2.0766	0.0082	0.0051
$[Cl$-∗-$ICl]^-$ (**47**: $D_{\infty h}$)	2.5514	2.5622	2.5568	0.0109	0.0054
$[Br$-∗-$IBr]^-$ (**48**: $D_{\infty h}$)	2.7091	2.7132	2.7114	0.0041	0.0023
Me_2FTe-∗-F (**49**: C_2)	1.9748	1.9835	1.9778	0.0087	0.0031
Me_2ClTe-∗-Cl (**50**: C_2)	2.4611	2.4672	2.4649	0.0061	0.0038
Me_2BrTe-∗-Br (**51**: C_2)	2.6266	2.6298	2.6288	0.0033	0.0022
Me_2ITe-∗-I (**52**: C_2)	2.8667	2.8705	2.8721	0.0038	0.0054
Me_2S^+-∗-I (**53**: C_s)	2.3600	2.3628	2.3643	0.0027	0.0042
Me_2Se^+-∗-I (**54**: C_s)	2.4737	2.4758	2.4789	0.0021	0.0052
Me_2Te^+-∗-F (**55**: C_s)	1.8635	1.8730	1.8655	0.0095	0.0021
Me_2Te^+-∗-Cl (**56**: C_s)	2.2728	2.2732	2.2739	0.0004	0.0011
Me_2Te^+-∗-Br (**57**: C_s)	2.4221	2.4208	2.4228	-0.0013	0.0007
Me_2Te^+-∗-I (**58**: C_s)	2.6365	2.6394	2.6372	0.0029	0.0007
I-∗-I (**59**: $D_{\infty h}$)	2.6545	2.6545	2.6545	0.0000	0.0000
CH_3-∗-F (**60**: C_{3v})	1.3884	1.3829	1.3852	-0.0055	-0.0033
CH_3-∗-I (**61**: C_{3v})	2.1123	2.1161	2.1196	0.0038	0.0073

[a] $\Delta r_o(X, Y)_{BSS-B'} = r_o(X, Y)_{BSS-B'} - r_o(X, Y)_{BSS-A'}$. [b] $\Delta r_o(X, Y)_{BSS-C} = r_o(X, Y)_{BSS-C} - r_o(X, Y)_{BSS-A'}$.

Table S3. QTAIM functions and QTAIM-DFA parameters for the standard interactions in **1**–**36**, evaluated under the MP2/BSS-A (aug-cc-pVTZ) condition, together with $\Delta\theta_p$ and those predicted nature.[a]

Species (X-∗-Y) (No: symmetry)	$\rho_b(\mathbf{r}_c)$ (au)	$c\nabla^2\rho_b(\mathbf{r}_c)$[b] (au)	$H_b(\mathbf{r}_c)$ (au)	R[c] (au)	θ[d] (°)	C_{ii}[e] (Å mdyn^{-1})	θ_p[f] (°)	$\kappa_{p:CIV}$[g] (au^{-1})	$\Delta\theta_p$[h] (°)	Predicted Nature
He-∗-HF (**1**: $C_{\infty v}$)	0.0033	0.0023	0.0013	0.0026	59.9	107.01	64.0	72.0	4.1	*p*-CS/vdW
Ne-∗-HF (**2**: $C_{\infty v}$)	0.0061	0.0038	0.0013	0.0041	71.0	43.86	78.9	15.6	7.9	*p*-CS/vdW
Ar-∗-HF (**3**: $C_{\infty v}$)	0.0088	0.0043	0.0015	0.0046	70.9	26.37	88.0	193.1	17.1	*p*-CS/vdW
Kr-∗-HF (**4**: $C_{\infty v}$)	0.0117	0.0047	0.0008	0.0048	80.0	15.46	111.9	264.6	31.9	*p*-CS/*t*-HB$_{nc}$
NN-∗-HF (**5**: $C_{\infty v}$)	0.0183	0.0079	0.0006	0.0079	85.6	9.78	132.8	175.1	47.2	*p*-CS/*t*-HB$_{nc}$
HF-∗-HF (**6**: C_s)	0.0251	0.0122	0.0000	0.0122	90.0	6.52	131.4	96.1	41.4	*p*-CS/*t*-HB$_{nc}$
HCN-∗-HF (**7**: $C_{\infty v}$)	0.0332	0.0101	-0.0054	0.0115	118.2	4.29	163.2	24.0	45.0	*r*-CS/CT-MC
H_2O-∗-HOH (**8**: C_s)	0.0247	0.0102	-0.0004	0.0102	92.3	6.63	136.9	117.4	44.6	*r*-CS/*t*-HB$_{wc}$
Me_2O-∗-HOH (**9**: C_s)	0.0322	0.0114	-0.0035	0.0119	107.0	5.21	155.7	49.6	48.7	*r*-CS/CT-MC
Me_2O-∗-Cl_2 (**10**: C_s)	0.0268	0.0130	0.0018	0.0131	82.0	6.04	103.3	68.1	21.3	*p*-CS/*t*-HB$_{nc}$
Me_2O-∗-Br_2 (**11**: C_s)	0.0334	0.0141	0.0000	0.0141	90.2	4.20	119.4	83.6	29.2	*r*-CS/*t*-HB$_{wc}$
Me_2S-∗-Cl_2 (**12**: C_s)	0.0521	0.0107	-0.0095	0.0143	131.6	6.60	172.1	24.7	40.5	*r*-CS/CT-MC
Me_2S-∗-Br_2 (**13**: C_s)	0.0521	0.0093	-0.0104	0.0139	138.2	2.94	173.2	23.0	35.0	*r*-CS/CT-MC
Me_2Se-∗-Cl_2 (**14**: C_s)	0.0633	0.0091	-0.0149	0.0175	148.6	2.68	179.4	9.5	30.8	*r*-CS/CT-MC
Me_2Se-∗-Br_2 (**15**: C_s)	0.0545	0.0078	-0.0116	0.0140	146.0	2.25	176.2	23.4	30.2	*r*-CS/CT-MC
$[Cl$-∗-$Cl_2]^-$ (**16**: $D_{\infty h}$)	0.0843	0.0125	-0.0246	0.0276	153.0	1.08	179.3	11.0	26.3	*r*-CS/CT-MC
$[Br$-∗-$Br_2]^-$ (**17**: $D_{\infty h}$)	0.0669	0.0084	-0.0173	0.0192	154.1	1.09	178.8	14.7	24.7	*r*-CS/CT-MC
$[Cl$-∗-$BrCl]^-$ (**18**: $D_{\infty h}$)	0.0762	0.0102	-0.0227	0.0249	155.8	1.02	180.5	8.7	24.7	*r*-CS/CT-TBP
$[Br$-∗-$ClBr]^-$ (**19**: $D_{\infty h}$)	0.0726	0.0104	-0.0187	0.0214	150.8	1.11	177.1	16.3	26.3	*r*-CS/CT-MC
Me_2ClS-∗-Cl (**20**: C_2)	0.0958	0.0041	-0.0370	0.0372	173.6	0.90	191.0	6.6	17.4	*r*-CS/CT-TBP
Me_2BrS-∗-Br (**21**: C_{2v})	0.0808	0.0047	-0.0260	0.0264	169.7	0.97	187.6	8.2	17.9	*r*-CS/CT-TBP
Me_2ClSe-∗-Cl (**22**: C_2)	0.0867	0.0047	-0.0342	0.0345	172.2	0.79	186.0	1.8	13.8	*r*-CS/CT-TBP
Me_2BrSe-∗-Br (**23**: C_2)	0.0755	0.0040	-0.0251	0.0254	170.9	0.88	186.4	6.0	15.5	*r*-CS/CT-TBP
Me_2S^+-∗-Cl (**24**: C_s)	0.1658	-0.0201	-0.1085	0.1103	190.5	0.31	197.7	0.6	7.2	SS/Cov-w
Me_2S^+-∗-Br (**25**: C_s)	0.1377	-0.0097	-0.0757	0.0763	187.3	0.36	194.0	0.6	6.7	SS/Cov-w
Me_2Se^+-∗-Cl (**26**: C_s)	0.1372	-0.0079	-0.0829	0.0833	185.5	0.33	186.1	4.2	0.6	SS/Cov-w
Me_2Se^+-∗-Br (**27**: C_s)	0.1189	-0.0067	-0.0606	0.0609	186.3	0.38	192.1	0.1	5.8	SS/Cov-w
Cl-∗-Cl (**28**: $D_{\infty h}$)	0.1582	-0.0049	-0.0872	0.0873	183.2	0.30	193.0	1.2	9.8	SS/Cov-w
Br-∗-Br (**29**: $D_{\infty h}$)	0.1132	-0.0013	-0.0517	0.0517	181.4	0.37	190.6	1.6	9.2	SS/Cov-w
CH_3-∗-Cl (**30**: C_{3v})	0.1848	-0.0333	-0.1352	0.1392	193.8	0.31	199.0	0.3	5.2	SS/Cov-w
CH_3-∗-Br (**31**: C_{3v})	0.1556	-0.0199	-0.0949	0.0969	191.8	0.35	196.3	0.1	4.5	SS/Cov-w
CH_3-∗-CH_3 (**32**: S_6)	0.2461	-0.0787	-0.2227	0.2362	199.5	0.23	202.0	0.0	2.5	SS/Cov-s
CH_2-∗-CH_2 (**33**: D_{2h})	0.3549	-0.1578	-0.4696	0.4954	198.6	0.11	200.0	0.1	1.4	SS/Cov-s
CH-∗-CH (**34**: $D_{\infty h}$)[i]										
CH_3-∗-H (**35**: T_d)	0.2845	-0.1388	-0.3298	0.3578	202.8	0.18	202.6	0.2	-0.2	SS/Cov-s
H-∗-H (**36**: $D_{\infty h}$)	0.2724	-0.1548	-0.3161	0.3520	206.1	0.17	206.3	0.0	0.2	SS/Cov-s

[a] Data are given for the interaction in question at the BCP, as shown by He-∗-HF, for example. [b] $c\nabla^2\rho_b(\mathbf{r}_c) = H_b(\mathbf{r}_c) - V_b(\mathbf{r}_c)/2$, where $c = \hbar^2/8m$. [c] $R = (x^2 + y^2)^{1/2}$, where $(x, y) = (H_b(\mathbf{r}_c) - V_b(\mathbf{r}_c)/2, H_b(\mathbf{r}_c))$. [d] $\theta = 90° - \tan^{-1}(y/x)$. [e] Compliance constants, see Equation (R1) in the main text. [f] $\theta_p = 90° - \tan^{-1}(dy/dx)$. [g] $\kappa_p = |d^2y/dx^2|/[1 + (dy/dx)^2]^{3/2}$. [h] $\Delta\theta_p = \theta_p - \theta$. [i] The (3, –3) attractor appeared at the center of the species.

Table S4. QTAIM functions and QTAIM-DFA parameters for the standard interactions in **1**–**36**, evaluated under the MP2/BSS-B (6-311++G(3df,3pd)) condition, together with $\Delta\theta_p$ and those predicted nature.[a]

Species (X-∗-Y) (No: symmetry)	$\rho_b(\boldsymbol{r}_c)$ (au)	$c\nabla^2\rho_b(\boldsymbol{r}_c)$[b] (au)	$H_b(\boldsymbol{r}_c)$ (au)	R[c] (au)	θ[d] (°)	C_{ii}[e] (Å mdyn^{-1})	θ_p[f] (°)	$\kappa_{p:CIV}$[g] (au^{-1})	$\Delta\theta_p$[h] (°)	Predicted Nature
He-∗-HF (**1**: $C_{\infty v}$)	0.0034	0.0022	0.0013	0.0025	59.9	106.55	57.2	8.2	-2.7	*p*-CS/vdW
Ne-∗-HF (**2**: $C_{\infty v}$)	0.0076	0.0050	0.0019	0.0054	69.2	28.15	84.4	85.3	15.2	*p*-CS/vdW
Ar-∗-HF (**3**: $C_{\infty v}$)	0.0083	0.0043	0.0020	0.0048	65.0	25.59	76.4	162.8	11.4	*p*-CS/vdW
Kr-∗-HF (**4**: $C_{\infty v}$)	0.0086	0.0040	0.0017	0.0043	66.5	25.74	80.4	220.6	13.9	*p*-CS/vdW
NN-∗-HF (**5**: $C_{\infty v}$)	0.0190	0.0087	0.0015	0.0088	80.0	8.62	126.8	237.8	46.8	*p*-CS/*t*-HB_{nc}
HF-∗-HF (**6**: C_s)	0.0250	0.0125	-0.0002	0.0125	90.8	5.93	128.2	107.1	37.4	*r*-CS/*t*-HB_{wc}
HCN-∗-HF (**7**: $C_{\infty v}$)	0.0337	0.0107	-0.0053	0.0120	116.1	4.20	168.5	22.2	52.4	*r*-CS/CT-MC
H_2O-∗-HOH (**8**: C_s)	0.0244	0.0106	0.0005	0.0107	87.3	6.37	123.7	159.2	36.4	*p*-CS/*t*-HB_{nc}
Me_2O-∗-HOH (**9**: C_s)	0.0314	0.0121	-0.0021	0.0123	99.8	5.21	148.9	98.9	49.1	*r*-CS/*t*-HB_{wc}
Me_2O-∗-Cl_2 (**10**: C_s)	0.0283	0.0126	0.0007	0.0126	86.7	5.10	96.5	30.6	9.8	*p*-CS/*t*-HB_{nc}
Me_2O-∗-Br_2 (**11**: C_s)	0.0302	0.0120	-0.0004	0.0120	91.7	4.73	106.6	46.7	14.9	*r*-CS/*t*-HB_{wc}
Me_2S-∗-Cl_2 (**12**: C_s)	0.0454	0.0108	-0.0055	0.0121	117.1	7.67	162.7	52.7	45.6	*r*-CS/CT-MC
Me_2S-∗-Br_2 (**13**: C_s)	0.0471	0.0092	-0.0076	0.0119	129.7	3.74	170.8	32.4	41.1	*r*-CS/CT-MC
Me_2Se-∗-Cl_2 (**14**: C_s)	0.0602	0.0091	-0.0129	0.0157	144.9	3.01	182.7	12.5	37.8	*r*-CS/CT-TBP
Me_2Se-∗-Br_2 (**15**: C_s)	0.0501	0.0075	-0.0098	0.0124	142.6	4.07	180.7	14.4	38.1	*r*-CS/CT-TBP
$[Cl$-∗-$Cl_2]^-$ (**16**: $D_{\infty h}$)	0.0836	0.0133	-0.0220	0.0257	149.0	1.18	181.6	10.7	32.6	*r*-CS/CT-TBP
$[Br$-∗-$Br_2]^-$ (**17**: $D_{\infty h}$)	0.0667	0.0075	-0.0179	0.0194	157.3	1.19	184.2	9.3	26.9	*r*-CS/CT-TBP
$[Cl$-∗-$BrCl]^-$ (**18**: $D_{\infty h}$)	0.0758	0.0097	-0.0224	0.0244	156.5	1.09	183.1	8.2	26.6	*r*-CS/CT-TBP
$[Br$-∗-$ClBr]^-$ (**19**: $D_{\infty h}$)	0.0721	0.0103	-0.0178	0.0205	150.0	1.20	181.5	12.1	31.5	*r*-CS/CT-TBP
Me_2ClS-∗-Cl (**20**: C_2)	0.0971	0.0044	-0.0369	0.0372	173.1	0.93	191.8	5.1	18.7	*r*-CS/CT-TBP
Me_2BrS-∗-Br (**21**: C_{2v})	0.0802	0.0047	-0.0253	0.0257	169.5	1.01	188.6	5.5	19.1	*r*-CS/CT-TBP
Me_2ClSe-∗-Cl (**22**: C_2)	0.0868	0.0047	-0.0341	0.0344	172.2	0.80	184.3	1.3	12.1	*r*-CS/CT-TBP
Me_2BrSe-∗-Br (**23**: C_2)	0.0749	0.0030	-0.0259	0.0260	173.3	0.92	187.3	2.6	14.0	*r*-CS/CT-TBP
Me_2S^+-∗-Cl (**24**: C_s)	0.1719	-0.0244	-0.1206	0.1230	191.5	0.30	198.2	0.4	6.7	SS/Cov-w
Me_2S^+-∗-Br (**25**: C_s)	0.1390	-0.0110	-0.0788	0.0796	187.9	0.36	193.8	0.3	5.9	SS/Cov-w
Me_2Se^+-∗-Cl (**26**: C_s)	0.1387	-0.0077	-0.0855	0.0858	185.2	0.32	185.4	1.5	0.2	SS/Cov-w
Me_2Se^+-∗-Br (**27**: C_s)	0.1192	-0.0082	-0.0627	0.0632	187.5	0.38	190.7	0.5	3.2	SS/Cov-w
Cl-∗-Cl (**28**: $D_{\infty h}$)	0.1641	-0.0087	-0.0985	0.0988	185.0	0.29	194.2	0.6	9.2	SS/Cov-w
Br-∗-Br (**29**: $D_{\infty h}$)	0.1154	-0.0044	-0.0574	0.0576	184.3	0.37	190.9	0.3	6.6	SS/Cov-w
CH_3-∗-Cl (**30**: C_{3v})	0.1907	-0.0376	-0.1468	0.1515	194.4	0.29	198.4	0.2	4.0	SS/Cov-w
CH_3-∗-Br (**31**: C_{3v})	0.1564	-0.0226	-0.0997	0.1022	192.8	0.35	195.9	0.3	3.1	SS/Cov-w
CH_3-∗-CH_3 (**32**: S_6)	0.2445	-0.0718	-0.2097	0.2217	198.9	0.23	201.1	0.1	2.2	SS/Cov-s
CH_2-∗-CH_2 (**33**: D_{2h})	0.3496	-0.1345	-0.4226	0.4435	197.7	0.11	199.6	0.0	1.9	SS/Cov-s
CH-∗-CH (**34**: $D_{\infty h}$)	0.4077	-0.1529	-0.6048	0.6238	194.2	0.06	196.0	0.0	1.8	SS/Cov-s
CH_3-∗-H (**35**: T_d)	0.2821	-0.1265	-0.3075	0.3325	202.4	0.18	202.3	0.1	-0.1	SS/Cov-s
H-∗-H (**36**: $D_{\infty h}$)	0.2733	-0.1544	-0.3154	0.3512	206.1	0.17	206.4	0.0	0.3	SS/Cov-s

[a] Data are given for the interaction in question at the BCP, as shown by He-∗-HF, for example. [b] $c\nabla^2\rho_b(\boldsymbol{r}_c) = H_b(\boldsymbol{r}_c) - V_b(\boldsymbol{r}_c)/2$, where $c = \hbar^2/8m$. [c] $R = (x^2 + y^2)^{1/2}$, where $(x, y) = (H_b(\boldsymbol{r}_c) - V_b(\boldsymbol{r}_c)/2, H_b(\boldsymbol{r}_c))$. [d] $\theta = 90° - \tan^{-1}(y/x)$. [e] Compliance constants, see Equation (R1) in the main text. [f] $\theta_p = 90° - \tan^{-1}(dy/dx)$. [g] $\kappa_p = |d^2y/dx^2|/[1 + (dy/dx)^2]^{3/2}$. [h] $\Delta\theta_p = \theta_p - \theta$.

Table S5. QTAIM functions and QTAIM-DFA parameters for the standard interactions in **1**–**36**, evaluated under the MP2/BSS-C (Sapporo-TZPsp) condition, together with $\Delta\theta_p$ and those predicted nature.[a]

Species (X-*-Y) (No: symmetry)	$\rho_b(\mathbf{r}_c)$ (au)	$c\nabla^2\rho_b(\mathbf{r}_c)$[b] (au)	$H_b(\mathbf{r}_c)$ (au)	R[c] (au)	θ[d] (°)	C_{ii}[e] (Å mdyn^{-1})	θ_p[f] (°)	$\kappa_{p:CIV}$[g] (au^{-1})	$\Delta\theta_p$[h] (°)	Predicted Nature
He-*-HF (**1**: $C_{\infty v}$)	0.0024	0.0018	0.0011	0.0020	58.9	210.48	62.3	69.8	3.5	*p*-CS/vdW
Ne-*-HF (**2**: $C_{\infty v}$)	0.0048	0.0030	0.0011	0.0032	70.3	75.94	77.8	16.0	7.4	*p*-CS/vdW
Ar-*-HF (**3**: $C_{\infty v}$)	0.0074	0.0037	0.0014	0.0040	69.1	40.89	83.4	157.2	14.3	*p*-CS/vdW
Kr-*-HF (**4**: $C_{\infty v}$)	0.0104	0.0043	0.0008	0.0044	78.9	21.74	106.2	211.4	27.2	*p*-CS/*t*-HB_{nc}
NN-*-HF (**5**: $C_{\infty v}$)	0.0183	0.0080	0.0008	0.0081	84.2	10.07	123.3	156.9	39.1	*p*-CS/*t*-HB_{nc}
HF-*-HF (**6**: C_s)	0.0246	0.0124	0.0009	0.0125	85.8	6.82	117.9	125.2	32.1	*p*-CS/*t*-HB_{nc}
HCN-*-HF (**7**: $C_{\infty v}$)	0.0336	0.0109	-0.0046	0.0118	113.0	4.27	159.0	49.1	46.1	*r*-CS/CT-MC
H_2O-*-HOH (**8**: C_s)	0.0242	0.0105	0.0004	0.0105	88.0	6.71	123.4	128.5	35.4	*p*-CS/*t*-HB_{nc}
Me_2O-*-HOH (**9**: C_s)	0.0315	0.0120	-0.0021	0.0122	99.8	5.33	145.2	89.4	45.4	*r*-CS/*t*-HB_{wc}
Me_2O-*-Cl_2 (**10**: C_s)	0.0240	0.0118	0.0023	0.0121	79.1	6.68	93.2	52.6	14.1	*p*-CS/*t*-HB_{nc}
Me_2O-*-Br_2 (**11**: C_s)	0.0302	0.0134	0.0013	0.0135	84.3	4.54	107.2	85.5	22.8	*p*-CS/*t*-HB_{nc}
Me_2S-*-Cl_2 (**12**: C_s)	0.0463	0.0108	-0.0060	0.0124	118.8	8.25	162.9	49.5	44.1	*r*-CS/CT-MC
Me_2S-*-Br_2 (**13**: C_s)	0.0495	0.0100	-0.0078	0.0127	127.8	3.17	167.4	38.2	39.6	*r*-CS/CT-MC
Me_2Se-*-Cl_2 (**14**: C_s)	0.0597	0.0101	-0.0114	0.0152	138.6	3.55	176.9	18.7	38.3	*r*-CS/CT-MC
Me_2Se-*-Br_2 (**15**: C_s)	0.0516	0.0089	-0.0088	0.0125	134.5	2.46	171.0	32.2	36.4	*r*-CS/CT-MC
$[Cl$-*-$Cl_2]^-$ (**16**: $D_{\infty h}$)	0.0837	0.0137	-0.0216	0.0255	147.6	1.11	178.4	14.6	30.8	*r*-CS/CT-MC
$[Br$-*-$Br_2]^-$ (**17**: $D_{\infty h}$)	0.0660	0.0098	-0.0145	0.0175	145.9	1.09	176.5	22.2	30.6	*r*-CS/CT-MC
$[Cl$-*-$BrCl]^-$ (**18**: $D_{\infty h}$)	0.0753	0.0114	-0.0201	0.0231	150.5	1.03	179.9	12.2	29.4	*r*-CS/CT-MC
$[Br$-*-$ClBr]^-$ (**19**: $D_{\infty h}$)	0.0717	0.0117	-0.0158	0.0197	143.6	1.12	175.5	22.7	31.9	*r*-CS/CT-MC
Me_2ClS-*-Cl (**20**: C_2)	0.0964	0.0046	-0.0362	0.0364	172.8	0.92	191.5	5.4	18.7	*r*-CS/CT-TBP
Me_2BrS-*-Br (**21**: C_{2v})	0.0804	0.0058	-0.0238	0.0245	166.4	0.98	187.4	10.1	21.0	*r*-CS/CT-TBP
Me_2ClSe-*-Cl (**22**: C_2)	0.0860	0.0053	-0.0325	0.0330	170.8	0.81	187.5	3.0	16.7	*r*-CS/CT-TBP
Me_2BrSe-*-Br (**23**: C_2)	0.0742	0.0053	-0.0225	0.0231	166.8	0.90	186.2	8.9	19.4	*r*-CS/CT-TBP
Me_2S^+-*-Cl (**24**: C_s)	0.1692	-0.0225	-0.1143	0.1165	191.1	0.30	197.9	0.3	6.8	SS/Cov-w
Me_2S^+-*-Br (**25**: C_s)	0.1389	-0.0103	-0.0771	0.0778	187.6	0.36	195.1	0.5	7.5	SS/Cov-w
Me_2Se^+-*-Cl (**26**: C_s)	0.1387	-0.0089	-0.0850	0.0854	185.9	0.32	186.0	4.4	0.0	SS/Cov-w
Me_2Se^+-*-Br (**27**: C_s)	0.1190	-0.0065	-0.0603	0.0607	186.2	0.38	193.5	0.1	7.3	SS/Cov-w
Cl-*-Cl (**28**: $D_{\infty h}$)	0.1606	-0.0056	-0.0895	0.0897	183.6	0.29	194.3	0.9	10.7	SS/Cov-w
Br-*-Br (**29**: $D_{\infty h}$)	0.1130	-0.0001	-0.0497	0.0497	180.1	0.37	191.8	1.8	11.7	SS/Cov-w
CH_3-*-Cl (**30**: C_{3v})	0.1855	-0.0338	-0.1362	0.1404	193.9	0.30	199.1	0.2	5.2	SS/Cov-w
CH_3-*-Br (**31**: C_{3v})	0.1554	-0.0198	-0.0945	0.0965	191.9	0.35	197.0	0.1	5.2	SS/Cov-w
CH_3-*-CH_3 (**32**: S_6)	0.2462	-0.0791	-0.2233	0.2369	199.5	0.23	201.8	0.0	2.3	SS/Cov-s
CH_2-*-CH_2 (**33**: D_{2h})	0.3545	-0.1527	-0.4608	0.4854	198.3	0.11	199.3	0.1	1.0	SS/Cov-s
CH-*-CH (**34**: $D_{\infty h}$)[i]	0.4109	-0.1608	-0.6278	0.6481	194.4	0.06	194.4	0.1	0.0	SS/Cov-s
CH_3-*-H (**35**: T_d)	0.2851	-0.1420	-0.3436	0.3718	202.5	0.18	201.5	0.4	-0.9	SS/Cov-s
H-*-H (**36**: $D_{\infty h}$)	0.2730	-0.1763	-0.3593	0.4002	206.1	0.16	206.4	0.0	0.2	SS/Cov-s

[a] Data are given for the interaction in question at the BCP, as shown by He-*-HF, for example. [b] $c\nabla^2\rho_b(\mathbf{r}_c) = H_b(\mathbf{r}_c) - V_b(\mathbf{r}_c)/2$, where $c = \hbar^2/8m$. [c] $R = (x^2 + y^2)^{1/2}$, where $(x, y) = (H_b(\mathbf{r}_c) - V_b(\mathbf{r}_c)/2, H_b(\mathbf{r}_c))$. [d] $\theta = 90° - \tan^{-1}(y/x)$. [e] Compliance constants, see Equation (R1) in the main text. [f] $\theta_p = 90° - \tan^{-1}(dy/dx)$. [g] $\kappa_p = |d^2y/dx^2|/[1 + (dy/dx)^2]^{3/2}$. [h] $\Delta\theta_p = \theta_p - \theta$. [i] Data from $w = 0$, ±0.025, and ±0.5 were employed, since the (3, –3) attractor appeared at the center of the perturbed structure in the case of $w = –0.1$.

Table S6. QTAIM functions and QTAIM-DFA parameters for the standard interactions in **37–61**, evaluated under the MP2/BSS-B (6-311++G(3df,3pd)) condition, together with $\Delta\theta_p$ and those predicted nature.[a]

Species (X-∗-Y) (No: symmetry)	$\rho_b(\boldsymbol{r}_c)$ (au)	$c\nabla^2\rho_b(\boldsymbol{r}_c)^b$ (au)	$H_b(\boldsymbol{r}_c)$ (au)	R^c (au)	θ^d (°)	$C_{ii}{}^e$ (Å mdyn^{-1})	$\theta_p{}^f$ (°)	$\kappa_{p:CIV}{}^g$ (au^{-1})	$\Delta\theta_p{}^h$ (°)	Predicted Nature
Xe-∗-HF (**37**: $C_{\infty v}$)	0.0108	0.0040	0.0013	0.0042	72.6	15.38	94.7	334.9	22.0	*p*-CS/*t*-HB$_{nc}$
Me_2O-∗-I_2 (**38**: C_s)	0.0302	0.0102	-0.0018	0.0103	99.9	4.65	121.0	75.2	21.1	*r*-CS/*t*-HB$_{wc}$
Me_2S-∗-I_2 (**39**: C_s)	0.0365	0.0069	-0.0052	0.0087	127.2	3.94	168.5	42.1	41.3	*r*-CS/CT-MC
Me_2Se-∗-I_2 (**40**: C_s)	0.0371	0.0061	-0.0056	0.0083	132.6	3.84	174.7	37.4	42.1	*r*-CS/CT-MC
Me_2Te-∗-F_2 (**41**: C_s)	0.0763	0.0246	-0.0177	0.0303	125.8	0.61	123.4	7.8	-2.3	*r*-CS/*t*-HB$_{wc}$
Me_2Te-∗-Cl_2 (**42**: C_s)	0.0687	0.0054	-0.0206	0.0213	165.2	1.26	184.4	0.4	19.2	*r*-CS/CT-TBP
Me_2Te-∗-Br_2 (**43**: C_s)	0.0574	0.0048	-0.0149	0.0157	162.1	1.81	186.3	5.5	24.1	*r*-CS/CT-TBP
Me_2Te-∗-I_2 (**44**: C_s)	0.0415	0.0045	-0.0079	0.0091	150.5	2.88	181.9	20.6	31.4	*r*-CS/CT-TBP
$[I$-∗-$I_2]^-$ (**45**: $D_{\infty h}$)	0.0508	0.0045	-0.0119	0.0128	159.1	1.42	183.8	14.0	24.6	*r*-CS/CT-TBP
$[F$-∗-$IF]^-$ (**46**: $D_{\infty h}$)	0.0909	0.0310	-0.0295	0.0428	133.5	0.52	119.6	12.6	-13.9	*r*-CS/*t*-HB$_{wc}$
$[Cl$-∗-$ICl]^-$ (**47**: $D_{\infty h}$)	0.0652	0.0080	-0.0199	0.0214	158.0	1.07	178.9	0.8	20.8	*r*-CS/CT-MC
$[Br$-∗-$IBr]^-$ (**48**: $D_{\infty h}$)	0.0590	0.0060	-0.0165	0.0176	159.9	1.21	182.8	4.8	22.9	*r*-CS/CT-TBP
Me_2FTe-∗-F (**49**: C_2)	0.1066	0.0566	-0.0298	0.0639	117.8	0.35	103.8	1.2	-14.0	*r*-CS/*t*-HB$_{wc}$
Me_2ClTe-∗-Cl (**50**: C_2)	0.0774	0.0078	-0.0303	0.0313	165.5	0.71	160.6	20.9	-4.9	*r*-CS/CT-MC
Me_2BrTe-∗-Br (**51**: C_2)	0.0697	0.0033	-0.0264	0.0266	172.8	0.83	177.3	11.9	4.5	*r*-CS/CT-MC
Me_2ITe-∗-I (**52**: C_2)	0.0596	0.0012	-0.0193	0.0193	176.5	1.01	187.7	1.5	11.2	*r*-CS/CT-TBP
Me_2S^+-∗-I (**53**: C_s)	0.1069	-0.0014	-0.0537	0.0537	181.5	0.44	179.5	7.3	-2.1	SS/Cov-w
Me_2Se^+-∗-I (**54**: C_s)	0.0983	-0.0048	-0.0474	0.0477	185.7	0.46	188.0	2.9	2.3	SS/Cov-w
Me_2Te^+-∗-F (**55**: C_s)	0.1362	0.0863	-0.0433	0.0965	116.7	0.21	107.2	2.0	-9.5	*r*-CS/*t*-HB$_{wc}$
Me_2Te^+-∗-Cl (**56**: C_s)	0.1108	0.0120	-0.0548	0.0561	167.7	0.33	145.1	8.3	-22.5	*r*-CS/*t*-HB$_{wc}$
Me_2Te^+-∗-Br (**57**: C_s)	0.1007	0.0004	-0.0507	0.0507	179.6	0.38	165.7	14.1	-13.9	*r*-CS/CT-MC
Me_2Te^+-∗-I (**58**: C_s)	0.0866	-0.0049	-0.0395	0.0398	187.1	0.48	189.5	3.4	2.4	SS/Cov-w
I-∗-I (**59**: $D_{\infty h}$)	0.0825	-0.0022	-0.0343	0.0344	183.7	0.48	190.9	0.5	7.2	SS/Cov-w
CH_3-∗-F (**60**: C_{3v})	0.2403	-0.0016	-0.3540	0.3540	180.3	0.19	154.5	1.3	-25.8	Cov-s
CH_3-∗-I (**61**: C_{3v})	0.1267	-0.0109	-0.0758	0.0766	188.2	0.40	180.7	9.0	-7.6	SS/Cov-w

[a] Data are given for the interaction in question at the BCP, as shown by Xe-∗-HF, for example. [b] $c\nabla^2\rho_b(\boldsymbol{r}_c) = H_b(\boldsymbol{r}_c) - V_b(\boldsymbol{r}_c)/2$, where $c = \hbar^2/8m$. [c] $R = (x^2 + y^2)^{1/2}$, where $(x, y) = (H_b(\boldsymbol{r}_c) - V_b(\boldsymbol{r}_c)/2, H_b(\boldsymbol{r}_c))$. [d] $\theta = 90° - \tan^{-1}(y/x)$. [e] Compliance constants, see Equation (R1) in the main text. [f] $\theta_p = 90° - \tan^{-1}(dy/dx)$. [g] $\kappa_p = |d^2y/dx^2|/[1 + (dy/dx)^2]^{3/2}$. [h] $\Delta\theta_p = \theta_p - \theta$.

Table S7. QTAIM functions and QTAIM-DFA parameters for the standard interactions in **37–61**, evaluated under the MP2/BSS-C (Sapporo-TZPsp) condition, together with $\Delta\theta_p$ and those predicted nature.[a]

Species (X-∗-Y) (No: symmetry)	$\rho_b(r_c)$ (au)	$c\nabla^2\rho_b(r_c)^b$ (au)	$H_b(r_c)$ (au)	R^c (au)	θ^d (°)	C_{ii}^e (Å mdyn^{-1})	θ_p^f (°)	$\kappa_{p:CIV}^g$ (au^{-1})	$\Delta\theta_p^h$ (°)	Predicted Nature
Xe-∗-HF (**37**: $C_{\infty v}$)	0.0105	0.0036	0.0006	0.0037	81.3	22.13	109.9	277.8	28.6	*p*-CS/*t*-HB$_{nc}$
Me_2O-∗-I_2 (**38**: C_s)	0.0280	0.0105	-0.0001	0.0105	90.3	4.79	122.6	121.9	32.2	*r*-CS/*t*-HB$_{wc}$
Me_2S-∗-I_2 (**39**: C_s)	0.0372	0.0070	-0.0055	0.0089	128.0	3.97	169.1	42.5	41.1	*r*-CS/CT-MC
Me_2Se-∗-I_2 (**40**: C_s)	0.0384	0.0062	-0.0059	0.0086	133.7	3.43	170.9	41.0	37.2	*r*-CS/CT-MC
Me_2Te-∗-F_2 (**41**: C_s)	0.0776	0.0250	-0.0201	0.0321	128.8	0.61	136.4	7.5	7.7	*r*-CS/*t*-HB$_{wc}$
Me_2Te-∗-Cl_2 (**42**: C_s)	0.0695	0.0053	-0.0215	0.0221	166.2	1.23	183.9	0.5	17.7	*r*-CS/CT-TBP
Me_2Te-∗-Br_2 (**43**: C_s)	0.0578	0.0055	-0.0143	0.0153	159.0	1.65	183.7	9.1	24.7	*r*-CS/CT-TBP
Me_2Te-∗-I_2 (**44**: C_s)	0.0416	0.0045	-0.0080	0.0092	150.8	2.95	182.0	20.4	31.2	*r*-CS/CT-TBP
$[I$-∗-$I_2]^-$ (**45**: $D_{\infty h}$)	0.0508	0.0045	-0.0119	0.0128	159.1	1.42	183.8	14.0	24.6	*r*-CS/CT-TBP
$[F$-∗-$IF]^-$ (**46**: $D_{\infty h}$)	0.0928	0.0323	-0.0327	0.0459	135.3	0.53	131.1	9.7	-4.2	*r*-CS/*t*-HB$_{wc}$
$[Cl$-∗-$ICl]^-$ (**47**: $D_{\infty h}$)	0.0663	0.0079	-0.0212	0.0226	159.7	1.02	177.9	1.4	18.2	*r*-CS/CT-MC
$[Br$-∗-$IBr]^-$ (**48**: $D_{\infty h}$)	0.0590	0.0064	-0.0158	0.0171	158.0	1.15	180.0	7.7	22.0	*r*-CS/CT-MC
Me_2FTe-∗-F (**49**: C_2)	0.1100	0.0583	-0.0368	0.0690	122.2	0.35	117.0	1.6	-5.3	*r*-CS/*t*-HB$_{wc}$
Me_2ClTe-∗-Cl (**50**: C_2)	0.0779	0.0078	-0.0311	0.0321	165.9	0.69	159.3	24.7	-6.7	*r*-CS/CT-MC
Me_2BrTe-∗-Br (**51**: C_2)	0.0695	0.0036	-0.0253	0.0256	171.8	0.80	175.8	12.3	4.0	*r*-CS/CT-MC
Me_2ITe-∗-I (**52**: C_2)	0.0594	0.0013	-0.0191	0.0191	176.2	1.02	187.6	1.9	11.4	*r*-CS/CT-TBP
Me_2S^+-∗-I (**53**: C_s)	0.1071	-0.0012	-0.0541	0.0542	181.3	0.44	178.4	8.1	-3.0	SS/Cov-w
Me_2Se^+-∗-I (**54**: C_s)	0.0967	-0.0032	-0.0444	0.0445	184.2	0.46	188.3	2.2	4.1	SS/Cov-w
Me_2Te^+-∗-F (**55**: C_s)	0.1424	0.0857	-0.0600	0.1046	125.0	0.21	120.9	1.0	-4.1	*r*-CS/*t*-HB$_{wc}$
Me_2Te^+-∗-Cl (**56**: C_s)	0.1106	0.0123	-0.0546	0.0560	167.3	0.33	142.7	8.9	-24.6	*r*-CS/*t*-HB$_{wc}$
Me_2Te^+-∗-Br (**57**: C_s)	0.0996	0.0012	-0.0478	0.0478	178.5	0.38	164.8	15.6	-13.7	*r*-CS/CT-MC
Me_2Te^+-∗-I (**58**: C_s)	0.0870	-0.0050	-0.0399	0.0402	187.2	0.47	189.6	3.6	2.4	SS/Cov-w
I-∗-I (**59**: $D_{\infty h}$)	0.0825	-0.0022	-0.0343	0.0344	183.7	0.48	190.9	0.5	7.2	SS/Cov-w
CH_3-∗-F (**60**: C_{3v})	0.2409	-0.0126	-0.3615	0.3617	182.0	0.19	164.3	1.6	-17.7	SS/Cov-s
CH_3-∗-I (**61**: C_{3v})	0.1243	-0.0086	-0.0689	0.0694	187.1	0.40	179.5	10.4	-7.6	SS/Cov-w

[a] Data are given for the interaction in question at the BCP, as shown by Xe-∗-HF, for example. [b] $c\nabla^2\rho_b(r_c) = H_b(r_c) - V_b(r_c)/2$, where $c = \hbar^2/8m$. [c] $R = (x^2 + y^2)^{1/2}$, where $(x, y) = (H_b(r_c) - V_b(r_c)/2, H_b(r_c))$. [d] $\theta = 90° - \tan^{-1}(y/x)$. [e] Compliance constants, see Equation (R1) in the main text. [f] $\theta_p = 90° - \tan^{-1}(dy/dx)$. [g] $\kappa_p = |d^2y/dx^2|/[1 + (dy/dx)^2]^{3/2}$. [h] $\Delta\theta_p = \theta_p - \theta$.

Table S8. QTAIM functions and QTAIM-DFA parameters for the standard interactions in [MeEE'Me]* and [HEE'H]* (E = S, Se, and Te; * = null, –, +, and/or 2+), evaluated under the RMP2/BSS-C (Sapporo-TZPsp) or UMP2/BSS-C condition, together with $\Delta\theta_p$ and those predicted nature.[a]

Species (symmetry.) (X-∗-Y) (No.)	$\rho_b(\mathbf{r}_c)$ (au)	$c\nabla^2\rho_b(\mathbf{r}_c)$[b] (au)	$H_b(\mathbf{r}_c)$ (au)	R[c] (au)	θ[d] (°)	C_{ii}[e] (Å mdyn^{-1})	θ_p[f] (°)	$\kappa_{p:CIV}$[g] (au^{-1})	$\Delta\theta_p$[h] (°)	Predicted Nature
MeSSMe (C_2)										
S-∗-S (1E_{Me}^0)	0.1551	-0.0204	-0.0979	0.0999	191.8	0.363	197.6	0.3	5.8	SS/Cov-w
S-∗-C	0.1831	-0.0374	-0.1300	0.1353	196.1	0.335	200.3	0.0	4.2	SS/Cov-w
MeSSeMe (C_1)										
S-∗-Se (2E_{Me}^0)	0.1287	-0.0107	-0.0726	0.0734	188.4	0.404	192.7	1.3	4.3	SS/Cov-w
S-∗-C	0.1818	-0.0367	-0.1281	0.1333	196.0	0.336	200.3	0.0	4.3	SS/Cov-w
Se-∗-C	0.1511	-0.0199	-0.0962	0.0982	191.7	0.374	190.3	3.7	-1.4	SS/Cov-w
MeSTeMe (C_1)										
S-∗-Te (3E_{Me}^0)	0.1018	0.0024	-0.0492	0.0493	177.2	0.436	163.0	14.2	-14.2	*r*-CS/CT-MC
S-∗-C	0.1786	-0.0349	-0.1239	0.1287	195.7	0.341	200.2	0.0	4.4	SS/Cov-w
Te-∗-C	0.1174	0.0013	-0.0615	0.0615	178.8	0.416	154.3	15.0	-24.5	*r*-CS/CT-MC
MeSeSeMe (C_2)										
Se-∗-Se (4E_{Me}^0)	0.1110	-0.0071	-0.0551	0.0556	187.3	0.444	194.1	0.4	6.8	SS/Cov-w
Se-∗-C	0.1502	-0.0197	-0.0950	0.0970	191.7	0.375	190.5	3.6	-1.2	SS/Cov-w
MeSeTeMe (C_1)										
Se-∗-Te (5E_{Me}^0)	0.0919	-0.0021	-0.0420	0.0421	182.8	0.482	179.1	9.6	-3.7	SS/Cov-w
Se-∗-C	0.1481	-0.0189	-0.0925	0.0944	191.5	0.381	190.8	3.5	-0.8	SS/Cov-w
Te-∗-C	0.1169	0.0012	-0.0610	0.0610	178.9	0.418	154.6	15.3	-24.3	*r*-CS/CT-MC
MeTeTeMe (C_2)										
Te-∗-Te (6E_{Me}^0)	0.0811	-0.0050	-0.0361	0.0365	187.8	0.541	190.5	1.9	2.7	SS/Cov-w
Te-∗-C	0.1156	0.0007	-0.0598	0.0598	179.3	0.425	155.8	16.3	-23.5	*r*-CS/CT-MC
[MeSSMe]$^-$ (C_1)										
S-∗-S (1E_{Me}^-)	0.0452	0.0079	-0.0064	0.0101	128.9	2.088	161.2	57.0	32.2	*r*-CS/CT-MC
S-∗-C	0.1769	-0.0336	-0.1255	0.1299	195.0	0.330	198.9	0.3	3.9	SS/Cov-w
[MeSSeMe]$^-$ (C_1)										
S-∗-Se (2E_{Me}^-)	0.0431	0.0071	-0.0060	0.0093	129.9	0.269	162.1	62.4	32.1	*r*-CS/CT-MC
S-∗-C	0.1760	-0.0330	-0.1244	0.1287	194.9	0.332	198.8	0.2	3.9	SS/Cov-w
Se-∗-C	0.1448	-0.0151	-0.0903	0.0916	189.5	0.373	186.7	4.6	-2.8	SS/Cov-w
[MeSTeMe]$^-$ (C_1)										
S-∗-Te (3E_{Me}^-)	0.0435	0.0050	-0.0085	0.0099	149.8	2.284	178.0	24.1	28.2	*r*-CS/CT-MC
S-∗-C	0.1749	-0.0324	-0.1227	0.1270	194.8	0.335	198.9	0.2	4.1	SS/Cov-w
Te-∗-C	0.1115	0.0036	-0.0559	0.0560	176.3	0.428	152.8	14.1	-23.5	*r*-CS/CT-MC
[MeSeSeMe]$^-$ (C_1)										
Se-∗-Se (4E_{Me}^-)	0.0407	0.0063	-0.0053	0.0083	130.2	2.063	160.7	73.6	30.6	*r*-CS/CT-MC
Se-∗-C	0.1441	-0.0148	-0.0895	0.0907	189.4	0.375	186.7	4.6	-2.7	SS/Cov-w
[MeSeTeMe]$^-$ (C_1)										
Se-∗-Te (5E_{Me}^-)	0.0397	0.0045	-0.0067	0.0081	146.4	2.258	175.3	38.6	28.9	*r*-CS/CT-MC
Se-∗-C	0.1432	-0.0144	-0.0884	0.0896	189.3	0.379	186.8	4.5	-2.5	SS/Cov-w
Te-∗-C	0.1111	0.0037	-0.0555	0.0556	176.2	0.429	152.8	14.2	-23.3	*r*-CS/CT-MC
[MeTeTeMe]$^-$ (C_2)										
Te-∗-Te (6E_{Me}^-)	0.0360	0.0032	-0.0061	0.0069	151.9	2.396	180.6	36.2	28.7	*r*-CS/CT-TBP
Te-∗-C	0.1105	0.0033	-0.0549	0.0550	176.5	0.431	154.0	14.6	-22.5	*r*-CS/CT-MC

[a] Data are given for the interaction in question at the BCP, as shown by S-∗-S, for example. [b] $c\nabla^2\rho_b(\mathbf{r}_c) = H_b(\mathbf{r}_c) - V_b(\mathbf{r}_c)/2$, where $c = \hbar^2/8m$. [c] $R = (x^2 + y^2)^{1/2}$, where $(x, y) = (H_b(\mathbf{r}_c) - V_b(\mathbf{r}_c)/2, H_b(\mathbf{r}_c))$. [d] $\theta = 90^\circ - \tan^{-1}(y/x)$. [e] Compliance constants, see Equation (R1) in the main text. [f] $\theta_p = 90^\circ - \tan^{-1}(dy/dx)$. [g] $\kappa_p = |d^2y/dx^2|/[1 + (dy/dx)^2]^{3/2}$.

(Table S8 continued)

Species (symmetry.) (X-∗-Y) (No.)	$\rho_b(\boldsymbol{r}_c)$ (au)	$c\nabla^2\rho_b(\boldsymbol{r}_c)^b$ (au)	$H_b(\boldsymbol{r}_c)$ (au)	R^c (au)	θ^d (°)	$C_{ii}{}^e$ (Å mdyn^{-1})	$\theta_p{}^f$ (°)	$\kappa_{p:CIV}{}^g$ (au^{-1})	$\Delta\theta_p{}^h$ (°)	Predicted Nature
$[MeSSMe]^+$ (C_{2h})										
S-∗-S ($1E_{Me}{}^+$)	0.1722	-0.0271	-0.1201	0.1231	192.7	0.289	198.5	0.2	5.8	SS/Cov-w
S-∗-C	0.1830	-0.0374	-0.1309	0.1362	196.0	0.369	200.6	0.2	4.6	SS/Cov-w
$[MeSSeMe]^+$ (C_1)										
S-∗-Se ($2E_{Me}{}^+$)	0.1434	-0.0130	-0.0912	0.0921	188.1	0.263	194.6	3.8	6.5	SS/Cov-w
S-∗-C	0.1811	-0.0364	-0.1284	0.1334	195.8	0.377	200.6	0.2	4.8	SS/Cov-w
Se-∗-C	0.1541	-0.0234	-0.0977	0.1004	193.5	0.396	194.9	2.4	1.4	SS/Cov-w
$[MeSTeMe]^+$ (C_1)										
S-∗-Te ($3E_{Me}{}^+$)	0.1125	0.0043	-0.0582	0.0584	175.8	0.313	160.3	5.6	-15.5	*r*-CS/CT-MC
S-∗-C	0.1752	-0.0332	-0.1200	0.1245	195.4	0.382	200.4	0.2	5.0	SS/Cov-w
Te-∗-C	0.1239	-0.0046	-0.0680	0.0682	183.8	0.420	160.7	15.3	-23.1	SS/Cov-w
$[MeSeSeMe]^+$ (C_2)										
Se-∗-Se ($4E_{Me}{}^+$)	0.1214	-0.0096	-0.0661	0.0668	188.3	0.372	194.9	0.2	6.6	SS/Cov-w
Se-∗-C	0.1527	-0.0227	-0.0960	0.0986	193.3	0.401	194.9	2.6	1.6	SS/Cov-w
$[MeSeTeMe]^+$ (C_1)										
Se-∗-Te ($5E_{Me}{}^+$)	0.1012	-0.0012	-0.0501	0.0501	181.3	0.323	177.4	2.2	-3.9	SS/Cov-w
Se-∗-C	0.1487	-0.0211	-0.0913	0.0937	193.0	0.417	195.2	2.1	2.2	SS/Cov-w
Te-∗-C	0.1221	-0.0041	-0.0663	0.0664	183.5	0.425	161.0	15.4	-22.5	SS/Cov-w
$[MeTeTeMe]^+$ (C_2)										
Te-∗-Te ($6E_{Me}{}^+$)	0.0855	-0.0058	-0.0398	0.0403	188.3	0.480	191.4	1.8	3.1	SS/Cov-w
Te-∗-C	0.1192	-0.0038	-0.0635	0.0636	183.5	0.439	161.2	18.1	-22.3	SS/Cov-w
$[MeSSMe]^{2+}$ (C_{2h})										
S-∗-S ($1E_{Me}{}^{2+}$)	0.1872	-0.0318	-0.1412	0.1448	192.7	0.293	198.2	0.1	5.5	SS/Cov-w
S-∗-C	0.1888	-0.0397	-0.1463	0.1516	195.2	0.444	200.3	0.2	5.1	SS/Cov-s
$[MeSSeMe]^{2+}$ (C_1)										
S-∗-Se ($2E_{Me}{}^{2+}$)	0.1514	-0.0144	-0.0998	0.1008	188.2	0.346	190.8	2.5	2.6	SS/Cov-w
S-∗-C	0.1860	-0.0382	-0.1415	0.1466	195.1	0.446	200.3	0.2	5.2	SS/Cov-w
Se-∗-C	0.1578	-0.0251	-0.1028	0.1058	193.7	0.460	197.4	1.2	3.7	SS/Cov-w
$[MeSTeMe]^{2+}$ (C_1)										
S-∗-Te ($3E_{Me}{}^{2+}$)	0.1156	0.0073	-0.0603	0.0608	173.1	0.370	161.8	10.8	-11.3	*r*-CS/CT-MC
S-∗-C	0.1781	-0.0343	-0.1296	0.1341	194.8	0.469	200.2	0.2	5.4	SS/Cov-w
Te-∗-C	0.1281	-0.0084	-0.0722	0.0727	186.7	0.468	167.3	17.5	-19.4	SS/Cov-w
$[MeSeSeMe]^{2+}$ (C_2)										
Se-∗-Se ($4E_{Me}{}^{2+}$)	0.1286	-0.0103	-0.0739	0.0746	188.0	0.402	194.6	0.0	6.6	SS/Cov-w
Se-∗-C	0.1558	-0.0242	-0.1001	0.1030	193.6	0.478	197.3	1.2	3.7	SS/Cov-w
$[MeSeTeMe]^{2+}$ (C_1)										
Se-∗-Te ($5E_{Me}{}^{2+}$)	0.1031	-0.0009	-0.0515	0.0515	181.0	0.435	177.8	8.6	-3.2	SS/Cov-w
Se-∗-C	0.1507	-0.0221	-0.0939	0.0965	193.2	0.495	197.3	1.1	4.1	SS/Cov-w
Te-∗-C	0.1262	-0.0081	-0.0704	0.0708	186.6	0.474	167.8	17.6	-18.8	SS/Cov-w
$[MeTeTeMe]^{2+}$ (C_2)										
Te-∗-Te ($6E_{Me}{}^{2+}$)	0.0887	-0.0057	-0.0424	0.0428	187.7	0.524	190.9	1.9	3.2	SS/Cov-w
Te-∗-C	0.1228	-0.0077	-0.0670	0.0675	186.5	0.492	169.2	17.9	-17.3	SS/Cov-w

[a] Data are given for the interaction in question at the BCP, as shown by He-∗-HF, for example. [b] $c\nabla^2\rho_b(\boldsymbol{r}_c) = H_b(\boldsymbol{r}_c) - V_b(\boldsymbol{r}_c)/2$, where $c = \hbar^2/8m$. [c] $R = (x^2 + y^2)^{1/2}$, where $(x, y) = (H_b(\boldsymbol{r}_c) - V_b(\boldsymbol{r}_c)/2, H_b(\boldsymbol{r}_c))$. [d] $\theta = 90° - \tan^{-1}(y/x)$. [e] Compliance constants, see Equation (R1) in the main text. [f] $\theta_p = 90° - \tan^{-1}(dy/dx)$. [g] $\kappa_p = |d^2y/dx^2|/[1 + (dy/dx)^2]^{3/2}$.

(Table S8 continued)

Species (symmetry.) (X-*-Y) (No.)	$\rho_b(\boldsymbol{r}_c)$ (au)	$c\nabla^2\rho_b(\boldsymbol{r}_c)^b$ (au)	$H_b(\boldsymbol{r}_c)$ (au)	R^c (au)	θ^d (°)	C_{ii}^e (Å mdyn^{-1})	θ_p^f (°)	$\kappa_{p:CIV}^g$ (au^{-1})	$\Delta\theta_p^h$ (°)	Predicted Nature
HSSH (C_2)										
S-*-S (1E_H^0)	0.1482	-0.0186	-0.0897	0.0916	191.7	0.369	197.8	0.3	6.1	SS/Cov-w
S-*-H	0.2232	-0.0821	-0.2225	0.2372	200.2	0.232	197.7	1.8	-2.5	SS/Cov-s
HSSeH (C_1)										
S-*-Se (2E_H^0)	0.1235	-0.0099	-0.0672	0.0679	188.4	0.410	193.1	1.2	4.7	SS/Cov-w
S-*-H	0.2227	-0.0812	-0.2227	0.2371	200.0	0.231	197.1	2.1	-2.9	SS/Cov-s
Se-*-H	0.1833	-0.0348	-0.1535	0.1574	192.8	0.270	178.9	4.3	-13.9	SS/Cov-s
HSTeH (C_1)										
S-*-Te (3E_H^0)	0.0981	0.0020	-0.0463	0.0464	177.5	0.443	163.1	14.2	-14.4	*r*-CS/CT-MC
S-*-H	0.2213	-0.0798	-0.2211	0.2351	199.8	0.229	196.8	2.2	-3.0	SS/Cov-s
Te-*-H	0.1367	0.0038	-0.0846	0.0847	177.4	0.337	156.5	2.3	-20.9	*r*-CS/CT-MC
HSeSeH (C_2)										
Se-*-Se (4E_H^0)	0.1067	-0.0065	-0.0511	0.0515	187.3	0.453	194.3	0.5	7.0	SS/Cov-w
Se-*-H	0.1830	-0.0349	-0.1531	0.1570	192.8	0.268	179.7	4.1	-13.1	SS/Cov-s
HSeTeH (C_1)										
Se-*-Te (5E_H^0)	0.0886	-0.0021	-0.0394	0.0395	183.0	0.493	179.7	10.3	-3.3	SS/Cov-w
Se-*-H	0.1823	-0.0335	-0.1521	0.1558	192.4	0.267	178.7	4.2	-13.7	SS/Cov-s
Te-*-H	0.1365	0.0039	-0.0844	0.0845	177.4	0.336	156.6	2.2	-20.8	*r*-CS/CT-MC
HTeTeH (C_2)										
Te-*-Te (6E_H^0)	0.0783	-0.0047	-0.0339	0.0342	187.9	0.558	190.9	1.8	3.0	SS/Cov-w
Te-*-H	0.1361	0.0032	-0.0841	0.0842	177.8	0.336	157.8	2.3	-20.0	*r*-CS/CT-MC
$[HSSH]^-$ (C_2)										
S-*-S (1E_H^-)	0.0422	0.0076	-0.0054	0.0093	125.3	2.131	158.5	64.7	33.2	*r*-CS/CT-MC
S-*-H[i]	0.2153	-0.0736	-0.2175	0.2296	198.7	0.228	195.0	2.3	-3.7	SS/Cov-s
$[HSSeH]^-$ (C_1)										
S-*-Se (2E_H^-)[i]	0.0401	0.0069	-0.0049	0.0085	125.4	2.087	158.4	73.2	33.0	*r*-CS/CT-MC
S-*-H	0.2145	-0.0726	-0.2178	0.2296	198.4	0.228	194.0	2.7	-4.4	SS/Cov-s
Se-*-H	0.1779	-0.0276	-0.1466	0.1491	190.7	0.265	177.7	3.8	-13.0	SS/Cov-w
$[HSTeH]^-$ (C_1)										
S-*-Te (3E_H^-)	0.0391	0.0051	-0.0065	0.0082	141.9	2.243	174.1	36.8	32.2	*r*-CS/CT-MC
S-*-H[i]	0.2138	-0.0719	-0.2167	0.2283	198.4	0.229	193.9	2.7	-4.5	SS/Cov-s
Te-*-H	0.1332	0.0072	-0.0803	0.0806	174.9	0.335	157.0	1.6	-17.9	*r*-CS/CT-MC
$[HSeSeH]^-$ (C_2)										
Se-*-Se (4E_H^-)	0.0377	0.0062	-0.0043	0.0075	124.9	2.099	155.9	88.7	31.0	*r*-CS/CT-MC
Se-*-H	0.1773	-0.0277	-0.1457	0.1483	190.8	0.266	178.6	3.6	-12.2	SS/Cov-w
$[HSeTeH]^-$ (C_1)										
Se-*-Te (5E_H^-)	0.0358	0.0046	-0.0051	0.0068	138.3	2.291	170.3	61.7	32.0	*r*-CS/CT-MC
Se-*-H	0.1768	-0.0267	-0.1450	0.1474	190.4	0.266	177.5	3.8	-12.9	SS/Cov-w
Te-*-H	0.0857	0.0073	-0.0799	0.0802	174.8	0.335	157.0	1.6	-17.8	*r*-CS/CT-MC
$[HTeTeH]^-$ (C_2)										
Te-*-Te (6E_H^-)	0.0317	0.0035	-0.0043	0.0055	141.3	2.520	174.8	59.0	33.5	*r*-CS/CT-MC
Te-*-H	0.1324	0.0063	-0.0797	0.0799	175.5	0.336	158.4	1.7	-17.1	*r*-CS/CT-MC

[a] Data are given for the interaction in question at the BCP, as shown by He-*-HF, for example. [b] $c\nabla^2\rho_b(\boldsymbol{r}_c) = H_b(\boldsymbol{r}_c) - V_b(\boldsymbol{r}_c)/2$, where $c = \hbar^2/8m$. [c] $R = (x^2 + y^2)^{1/2}$, where $(x, y) = (H_b(\boldsymbol{r}_c) - V_b(\boldsymbol{r}_c)/2, H_b(\boldsymbol{r}_c))$. [d] $\theta = 90° - \tan^{-1}(y/x)$. [e] Compliance constants, see Equation (R1) in the main text. [f] $\theta_p = 90° - \tan^{-1}(dy/dx)$. [g] $\kappa_p = |d^2y/dx^2|/[1 + (dy/dx)^2]^{3/2}$. [h] $\Delta\theta_p = \theta_p - \theta$. [i] Data from $w = 0, \pm0.025$, and ±0.5 were employed, since plot of $H_b(\boldsymbol{r}_c)$ versus $H_b(\boldsymbol{r}_c) - V_b(\boldsymbol{r}_c)/2$ gave poor correlation.

(Table S8 continued)

Species (symmetry.) (X-*-Y) (No.)	$\rho_b(\boldsymbol{r}_c)$ (au)	$c\nabla^2\rho_b(\boldsymbol{r}_c)^b$ (au)	$H_b(\boldsymbol{r}_c)$ (au)	R^c (au)	θ^d (°)	C_{ii}^e (Å mdyn^{-1})	θ_p^f (°)	$\kappa_{p:CIV}^g$ (au^{-1})	$\Delta\theta_p^h$ (°)	Predicted Nature
$[HSSH]^{2+}$ (C_{2h})										
S-*-S (1E_H^{2+})	0.1838	-0.0306	-0.1362	0.1396	192.7	0.293	198.3	0.1	5.6	SS/Cov-w
S-*-H	0.2187	-0.0912	-0.2098	0.2288	203.5	0.288	203.5	0.2	0.0	SS/Cov-s
$[HSSeH]^{2+}$ (C_s)										
S-*-Se (2E_H^{2+})	0.1483	-0.0141	-0.0962	0.0972	188.3	0.344	191.5	2.2	3.2	SS/Cov-w
S-*-H	0.2191	-0.0896	-0.2091	0.2275	203.2	0.280	203.2	0.3	0.0	SS/Cov-s
Se-*-H	0.1861	-0.0566	-0.1548	0.1648	200.1	0.317	193.2	3.6	-6.9	SS/Cov-s
$[HSTeH]^{2+}$ (C_s)										
S-*-Te (3E_H^{2+})	0.1143	0.0067	-0.0593	0.0597	173.6	0.348	163.9	10.4	-9.7	*r*-CS/CT-MC
S-*-H	0.2188	-0.0868	-0.2076	0.2250	202.7	0.270	202.5	0.4	0.2	SS/Cov-s
Te-*-H	0.1437	-0.0141	-0.0959	0.0969	188.4	0.366	161.6	6.7	-26.8	SS/Cov-w
$[HSeSeH]^{2+}$ (C_{2h})										
Se-*-Se (4E_H^{2+})	0.1249	-0.0097	-0.0698	0.0704	187.9	0.417	194.7	0.1	6.8	SS/Cov-w
Se-*-H	0.1857	-0.0546	-0.1543	0.1637	199.5	0.311	191.9	3.8	-7.6	SS/Cov-s
$[HSeTeH]^{2+}$ (C_s)										
Se-*-Te (5E_H^{2+})	0.1005	-0.0010	-0.0493	0.0493	181.2	0.432	179.6	8.7	-1.6	SS/Cov-w
Se-*-H	0.1845	-0.0515	-0.1531	0.1616	198.6	0.303	190.0	4.1	-8.6	SS/Cov-s
Te-*-H	0.1427	-0.0123	-0.0945	0.0953	187.4	0.362	161.1	6.2	-26.3	SS/Cov-w
$[HTeTeH]^{2+}$ (C_{2h})										
Te-*-Te (6E_H^{2+})	0.0856	-0.0053	-0.0396	0.0399	187.6	0.553	191.3	1.6	3.7	SS/Cov-w
Te-*-H	0.1412	-0.0099	-0.0923	0.0929	186.1	0.359	160.5	6.6	-25.6	SS/Cov-w

[a] Data are given for the interaction in question at the BCP, as shown by He-*-HF, for example. [b] $c\nabla^2\rho_b(\boldsymbol{r}_c) = H_b(\boldsymbol{r}_c) - V_b(\boldsymbol{r}_c)/2$, where $c = \hbar^2/8m$. [c] $R = (x^2 + y^2)^{1/2}$, where $(x, y) = (H_b(\boldsymbol{r}_c) - V_b(\boldsymbol{r}_c)/2, H_b(\boldsymbol{r}_c))$. [d] $\theta = 90° - \tan^{-1}(y/x)$. [e] Compliance constants, see Equation (R1) in the main text. [f] $\theta_p = 90° - \tan^{-1}(dy/dx)$. [g] $\kappa_p = |d^2y/dx^2|/[1 + (dy/dx)^2]^{3/2}$.

Table S9. QTAIM functions and QTAIM-DFA parameters for N-∗-Br for wide range of the interaction distances around TS [MeI–N(Tf)-∗-BrMe] (Tf: SO_2CF_3) by employing perturbed structures generated with IRC calculation, evaluated under the MP2/BSS-C' condition, together with $\Delta\theta_p$.[a,b]

w^c	$\rho_b(\boldsymbol{r}_c)$ (au)	$c\nabla^2\rho_b(\boldsymbol{r}_c)^d$ (au)	$H_b(\boldsymbol{r}_c)$ (au)	R^e (au)	θ^f (°)	$\theta_p{}^g$ (°)	$\kappa_p{}^h$ (au^{-1})	$\Delta\theta_p{}^i$ (°)
-0.601	0.1623	-0.0007	-0.0946	0.0946	180.4	194.0	0.3	13.6
-0.558	0.1550	0.0016	-0.0855	0.0856	178.9	194.1	0.0	15.2
-0.504	0.1463	0.0041	-0.0753	0.0755	176.9	194.0	0.3	17.2
-0.454	0.1388	0.0062	-0.0671	0.0674	174.7	193.8	0.6	19.1
-0.400	0.1310	0.0081	-0.0591	0.0597	172.2	193.5	0.9	21.3
-0.361	0.1256	0.0094	-0.0539	0.0547	170.2	193.2	1.2	23.0
-0.320	0.1203	0.0105	-0.0490	0.0501	167.9	192.8	1.4	24.9
-0.256	0.1123	0.0120	-0.0421	0.0438	164.1	192.2	1.9	28.1
-0.201	0.1059	0.0131	-0.0370	0.0392	160.5	191.6	2.5	31.1
-0.151	0.1003	0.0139	-0.0328	0.0356	157.0	190.9	3.2	34.0
-0.104	0.0954	0.0146	-0.0293	0.0327	153.5	190.3	4.3	36.8
-0.052	0.0902	0.0152	-0.0258	0.0299	149.5	189.4	5.1	39.9
0.000	0.0852	0.0157	-0.0226	0.0275	145.2	188.4	7.0	43.1
0.048	0.0809	0.0161	-0.0200	0.0256	141.2	187.2	9.2	45.9
0.101	0.0763	0.0163	-0.0174	0.0239	136.9	185.7	12.5	48.8
0.148	0.0724	0.0165	-0.0153	0.0226	132.9	184.0	16.3	51.1
0.201	0.0683	0.0166	-0.0133	0.0213	128.6	181.8	21.8	53.2
0.240	0.0654	0.0167	-0.0119	0.0205	125.5	180.0	26.7	54.5
0.310	0.0604	0.0166	-0.0097	0.0192	120.2	176.0	37.4	55.8
0.357	0.0574	0.0165	-0.0084	0.0185	117.0	173.0	46.3	56.0
0.403	0.0545	0.0163	-0.0072	0.0178	114.0	169.8	55.8	55.8
0.447	0.0518	0.0161	-0.0062	0.0172	111.2	166.3	66.8	55.1
0.492	0.0493	0.0158	-0.0053	0.0167	108.6	162.5	77.7	53.9
0.555	0.0459	0.0154	-0.0042	0.0160	105.3	156.7	93.6	51.4
0.597	0.0438	0.0151	-0.0035	0.0155	103.2	152.8	109.0	49.6
0.657	0.0410	0.0146	-0.0027	0.0149	100.5	146.9	119.3	46.4
0.695	0.0394	0.0143	-0.0023	0.0145	99.0	143.3	122.5	44.3
0.751	0.0371	0.0138	-0.0017	0.0139	96.8	137.9	126.4	41.1
0.803	0.0351	0.0133	-0.0012	0.0134	95.0	133.1	127.6	38.1
0.852	0.0333	0.0129	-0.0008	0.0129	93.4	128.8	125.7	35.4
0.899	0.0317	0.0124	-0.0004	0.0124	92.0	124.9	123.1	32.9
0.944	0.0303	0.0120	-0.0002	0.0120	90.7	121.3	119.2	30.6
1.001	0.0285	0.0114	0.0002	0.0114	89.2	117.3	114.8	28.0
1.056	0.0270	0.0109	0.0004	0.0109	87.9	113.6	113.1	25.7
1.096	0.0259	0.0105	0.0006	0.0106	87.0	110.9	111.0	23.9
1.147	0.0246	0.0101	0.0007	0.0101	85.9	107.8	104.9	22.0
1.197	0.0234	0.0096	0.0009	0.0096	84.9	105.0	101.5	20.1
1.247	0.0223	0.0092	0.0010	0.0092	84.0	102.5	98.1	18.5
1.295	0.0213	0.0088	0.0010	0.0088	83.2	100.1	94.7	17.0
1.356	0.0201	0.0083	0.0011	0.0083	82.2	97.4	90.4	15.2

[a] BSS-C': the Sapporo-TZP+sp for Br, I, and N with the 6-311+G(d,p) for C, H, F, O, and S. [b] Data are given for the interaction in question at the BCP. [c] w in $r = r_o + wa_o$ for TS [MeI–N(Tf)-∗-BrMe]. [d] $c\nabla^2\rho_b(\boldsymbol{r}_c) = H_b(\boldsymbol{r}_c) - V_b(\boldsymbol{r}_c)/2$, where $c = \hbar^2/8m$. [e] $R = (x^2 + y^2)^{1/2}$, where $(x, y) = (H_b(\boldsymbol{r}_c) - V_b(\boldsymbol{r}_c)/2, H_b(\boldsymbol{r}_c))$. [f] $\theta = 90° - \tan^{-1}(y/x)$. [g] $\theta_p = 90° - \tan^{-1}(dy/dx)$. [h] $\kappa_p = |d^2y/dx^2|/[1 + (dy/dx)^2]^{3/2}$. [i] $\Delta\theta_p = \theta_p - \theta$.

Table S10. QTAIM functions and QTAIM-DFA parameters for N-∗-I for wide range of the interaction distances around TS [MeI-∗-N(Tf)–BrMe] (Tf: SO_2CF_3) by employing perturbed structures generated with IRC calculation, evaluated under the MP2/BSS-C' condition, together with $\Delta\theta_p$.[a,b]

w^c	$\rho_b(\boldsymbol{r}_c)$ (au)	$c\nabla^2\rho_b(\boldsymbol{r}_c)^d$ (au)	$H_b(\boldsymbol{r}_c)$ (au)	R^e (au)	θ^f (°)	$\theta_p{}^g$ (°)	$\kappa_p{}^h$ (au^{-1})	$\Delta\theta_p{}^i$ (°)
-0.751	0.1275	0.0070	-0.0699	0.0703	174.2	168.4	11.6	-5.8
-0.705	0.1222	0.0062	-0.0647	0.0650	174.6	172.2	12.0	-2.4
-0.660	0.1172	0.0056	-0.0599	0.0602	174.6	175.5	11.4	0.9
-0.607	0.1117	0.0054	-0.0546	0.0549	174.4	178.8	10.3	4.4
-0.562	0.1073	0.0054	-0.0504	0.0507	173.9	181.1	9.2	7.2
-0.513	0.1026	0.0055	-0.0461	0.0464	173.1	183.3	8.3	10.2
-0.460	0.0978	0.0059	-0.0417	0.0421	172.0	185.3	7.4	13.3
-0.404	0.0929	0.0063	-0.0374	0.0379	170.4	187.0	6.8	16.6
-0.365	0.0896	0.0067	-0.0346	0.0352	169.0	188.0	7.4	19.0
-0.304	0.0846	0.0073	-0.0305	0.0313	166.4	189.6	6.5	23.1
-0.262	0.0813	0.0078	-0.0279	0.0290	164.4	190.4	6.0	26.0
-0.220	0.0780	0.0083	-0.0254	0.0267	161.9	191.2	5.3	29.2
-0.154	0.0731	0.0090	-0.0218	0.0236	157.5	192.2	4.1	34.6
-0.101	0.0693	0.0096	-0.0192	0.0215	153.5	192.7	2.5	39.2
-0.053	0.0659	0.0101	-0.0170	0.0198	149.3	192.9	0.2	43.6
0.000	0.0623	0.0106	-0.0148	0.0182	144.4	192.8	3.6	48.4
0.049	0.0591	0.0110	-0.0129	0.0170	139.6	192.3	7.4	52.7
0.101	0.0558	0.0114	-0.0111	0.0159	134.3	191.2	13.6	56.9
0.148	0.0529	0.0117	-0.0096	0.0151	129.5	189.8	21.4	60.3
0.197	0.0502	0.0119	-0.0083	0.0145	124.8	187.9	31.4	63.1
0.259	0.0468	0.0121	-0.0067	0.0138	119.0	184.4	49.1	65.4
0.300	0.0447	0.0121	-0.0058	0.0134	115.5	181.6	63.8	66.1
0.359	0.0418	0.0121	-0.0046	0.0129	110.9	176.7	89.6	65.8
0.397	0.0401	0.0120	-0.0039	0.0127	108.2	173.1	110.3	64.9
0.451	0.0379	0.0119	-0.0031	0.0123	104.7	167.3	139.7	62.6
0.501	0.0359	0.0117	-0.0024	0.0120	101.8	161.3	169.0	59.5
0.547	0.0342	0.0115	-0.0019	0.0116	99.4	155.5	192.8	56.1
0.603	0.0323	0.0112	-0.0013	0.0113	96.8	148.1	211.3	51.4
0.655	0.0307	0.0109	-0.0009	0.0109	94.6	141.5	234.6	46.9
0.702	0.0293	0.0105	-0.0005	0.0106	92.8	135.7	215.3	42.9

[a] BSS-C': the Sapporo-TZP+sp for Br, I, and N with the 6-311+G(d,p) for C, H, F, O, and S. [b] Data are given for the interaction in question at the BCP. [c] w in $r = r_o + wa_o$ for TS [MeI-∗-N(Tf)–BrMe]. [d] $c\nabla^2\rho_b(\boldsymbol{r}_c) = H_b(\boldsymbol{r}_c) - V_b(\boldsymbol{r}_c)/2$, where $c = \hbar^2/8m$. [e] $R = (x^2 + y^2)^{1/2}$, where $(x, y) = (H_b(\boldsymbol{r}_c) - V_b(\boldsymbol{r}_c)/2, H_b(\boldsymbol{r}_c))$. [f] $\theta = 90° - \tan^{-1}(y/x)$. [g] $\theta_p = 90° - \tan^{-1}(dy/dx)$. [h] $\kappa_p = |d^2y/dx^2|/[1 + (dy/dx)^2]^{3/2}$. [i] $\Delta\theta_p = \theta_p - \theta$.

Table S11. QTAIM functions and QTAIM-DFA parameters for N-∗-^{A}Br for wide range of the interaction distances around TS [Me^{A}Br–N(Tf)-∗-^{B}BrMe] (Tf: SO_2CF_3) by employing perturbed structures generated with IRC calculation, evaluated under the MP2/BSS-C' condition, together with $\Delta\theta_p$.[a,b]

w^c	$\rho_b(\boldsymbol{r}_c)$ (au)	$c\nabla^2\rho_b(\boldsymbol{r}_c)^d$ (au)	$H_b(\boldsymbol{r}_c)$ (au)	R^e (au)	θ^f (°)	$\theta_p{}^g$ (°)	$\kappa_p{}^h$ (au^{-1})	$\Delta\theta_p{}^i$ (°)
-0.750	0.1660	-0.0021	-0.0991	0.0992	181.2	193.7	0.2	12.4
-0.700	0.1567	0.0008	-0.0873	0.0873	179.5	193.8	0.1	14.3
-0.650	0.1488	0.0030	-0.0780	0.0781	177.8	193.7	0.4	15.9
-0.600	0.1418	0.0049	-0.0702	0.0704	176.0	193.4	0.7	17.4
-0.550	0.1345	0.0067	-0.0624	0.0628	173.8	193.1	1.0	19.2
-0.500	0.1267	0.0085	-0.0548	0.0555	171.2	192.6	1.3	21.4
-0.450	0.1214	0.0096	-0.0499	0.0508	169.1	192.2	1.5	23.1
-0.400	0.1135	0.0110	-0.0430	0.0444	165.6	191.6	1.7	25.9
-0.350	0.1083	0.0119	-0.0388	0.0406	163.0	191.1	1.9	28.2
-0.300	0.1032	0.0126	-0.0348	0.0371	160.1	190.7	2.2	30.6
-0.250	0.0983	0.0133	-0.0312	0.0339	156.9	190.2	2.6	33.3
-0.200	0.0917	0.0141	-0.0268	0.0302	152.2	189.5	3.4	37.3
-0.150	0.0871	0.0146	-0.0238	0.0279	148.5	188.9	4.7	40.4
-0.100	0.0821	0.0150	-0.0208	0.0256	144.1	188.1	6.3	44.0
-0.050	0.0779	0.0153	-0.0184	0.0239	140.1	187.3	8.1	47.2
0.000	0.0734	0.0156	-0.0159	0.0223	135.5	186.0	11.5	50.5
0.050	0.0694	0.0158	-0.0139	0.0211	131.3	184.4	15.4	53.1
0.100	0.0654	0.0159	-0.0120	0.0199	126.9	182.6	21.2	55.7
0.150	0.0619	0.0160	-0.0104	0.0191	123.1	180.4	28.7	57.4
0.200	0.0583	0.0160	-0.0089	0.0183	119.1	177.6	38.4	58.6
0.250	0.0551	0.0159	-0.0075	0.0176	115.4	174.4	49.9	58.9
0.300	0.0523	0.0157	-0.0065	0.0170	112.4	171.0	62.6	58.7
0.350	0.0496	0.0156	-0.0055	0.0165	109.5	167.3	75.9	57.8
0.400	0.0461	0.0152	-0.0043	0.0158	105.8	161.3	96.6	55.5
0.450	0.0440	0.0150	-0.0036	0.0154	103.6	157.2	109.8	53.5
0.500	0.0411	0.0145	-0.0028	0.0148	100.8	150.8	135.2	50.0
0.550	0.0395	0.0143	-0.0023	0.0144	99.1	146.9	138.8	47.8
0.600	0.0372	0.0138	-0.0017	0.0139	97.0	141.2	143.9	44.2
0.650	0.0353	0.0134	-0.0012	0.0134	95.1	136.0	146.6	40.9
0.700	0.0335	0.0130	-0.0008	0.0130	93.5	131.3	143.7	37.8
0.750	0.0315	0.0124	-0.0004	0.0124	91.6	125.8	138.9	34.1
0.800	0.0302	0.0120	-0.0001	0.0120	90.4	122.1	133.7	31.7
0.850	0.0285	0.0115	0.0002	0.0115	89.0	117.8	126.1	28.8
0.900	0.0271	0.0110	0.0005	0.0110	87.7	114.0	118.6	26.3
0.950	0.0257	0.0105	0.0006	0.0105	86.5	110.6	111.0	24.1
1.000	0.0244	0.0100	0.0008	0.0101	85.4	107.5	104.1	22.1
1.050	0.0232	0.0096	0.0009	0.0096	84.4	104.8	98.0	20.4
1.100	0.0221	0.0091	0.0010	0.0092	83.5	102.3	92.8	18.9
1.150	0.0210	0.0087	0.0011	0.0088	82.6	100.1	88.5	17.5
1.200	0.0200	0.0083	0.0012	0.0084	81.8	98.0	85.1	16.2
1.250	0.0191	0.0079	0.0012	0.0080	81.0	96.1	82.4	15.1
1.300	0.0182	0.0075	0.0013	0.0076	80.3	94.3	80.3	14.0
1.350	0.0173	0.0072	0.0013	0.0073	79.7	92.7	78.6	13.0
1.400	0.0163	0.0067	0.0013	0.0069	78.9	90.7	76.8	11.8
1.450	0.0156	0.0064	0.0013	0.0065	78.3	89.3	75.6	11.0

[a] BSS-C': the Sapporo-TZP+sp for Br and N with the 6-311+G(d,p) for C, H, F, O, and S. [b] Data are given for the interaction in question at the BCP. [c] w in $r = r_o + wa_o$ for TS [Me^{A}Br–N(Tf)-∗-^{B}BrMe]. [d] $c\nabla^2\rho_b(\boldsymbol{r}_c) = H_b(\boldsymbol{r}_c) - V_b(\boldsymbol{r}_c)/2$, where $c = \hbar^2/8m$. [e] $R = (x^2 + y^2)^{1/2}$, where $(x, y) = (H_b(\boldsymbol{r}_c) - V_b(\boldsymbol{r}_c)/2, H_b(\boldsymbol{r}_c))$. [f] $\theta = 90° - \tan^{-1}(y/x)$. [g] $\theta_p = 90° - \tan^{-1}(dy/dx)$. [h] $\kappa_p = |d^2y/dx^2|/[1 + (dy/dx)^2]^{3/2}$. [i] $\Delta\theta_p = \theta_p - \theta$.

Table S12. QTAIM functions and QTAIM-DFA parameters for N-∗-^{B}Br for wide range of the interaction distances around TS [Me^{A}Br–N(Tf)-∗-^{B}BrMe] (Tf: SO_2CF_3) by employing perturbed structures generated with IRC calculation, evaluated under the MP2/BSS-C' condition, together with $\Delta\theta_p$.[a,b]

w^c	$\rho_b(\boldsymbol{r}_c)$ (au)	$c\nabla^2\rho_b(\boldsymbol{r}_c)^d$ (au)	$H_b(\boldsymbol{r}_c)$ (au)	R^e (au)	θ^f (°)	$\theta_p{}^g$ (°)	$\kappa_p{}^h$ (au^{-1})	$\Delta\theta_p{}^i$ (°)
-0.650	0.1528	0.0005	-0.0833	0.0833	179.7	194.3	0.4	14.6
-0.600	0.1446	0.0029	-0.0739	0.0739	177.8	194.1	0.6	16.3
-0.550	0.1380	0.0046	-0.0667	0.0669	176.0	193.8	0.9	17.8
-0.500	0.1304	0.0065	-0.0590	0.0594	173.7	193.3	1.2	19.6
-0.450	0.1242	0.0079	-0.0530	0.0536	171.5	192.9	1.4	21.4
-0.400	0.1176	0.0092	-0.0471	0.0480	168.9	192.4	1.5	23.5
-0.350	0.1130	0.0101	-0.0432	0.0443	166.8	192.0	1.6	25.2
-0.300	0.1061	0.0113	-0.0376	0.0392	163.3	191.5	1.8	28.2
-0.250	0.1015	0.0120	-0.0341	0.0361	160.7	191.1	2.0	30.5
-0.200	0.0947	0.0129	-0.0292	0.0319	156.2	190.5	2.3	34.4
-0.150	0.0898	0.0135	-0.0260	0.0293	152.6	190.1	3.0	37.5
-0.100	0.0850	0.0140	-0.0230	0.0269	148.6	189.6	4.3	40.9
-0.050	0.0804	0.0144	-0.0202	0.0248	144.4	188.9	6.0	44.5
0.000	0.0742	0.0149	-0.0168	0.0225	138.3	187.5	9.4	49.1
0.050	0.0718	0.0151	-0.0155	0.0217	135.8	187.0	10.9	51.2
0.100	0.0677	0.0153	-0.0135	0.0204	131.3	185.5	15.6	54.1
0.150	0.0642	0.0155	-0.0118	0.0195	127.4	183.8	21.7	56.4
0.200	0.0605	0.0155	-0.0102	0.0186	123.2	181.5	30.0	58.3
0.250	0.0565	0.0155	-0.0085	0.0177	118.6	178.2	42.3	59.5
0.300	0.0536	0.0155	-0.0073	0.0171	115.3	175.1	54.3	59.7
0.350	0.0509	0.0154	-0.0063	0.0166	112.3	171.6	68.1	59.3
0.400	0.0483	0.0152	-0.0054	0.0161	109.6	167.7	83.0	58.2
0.450	0.0449	0.0149	-0.0042	0.0155	105.9	161.4	104.7	55.5
0.500	0.0428	0.0146	-0.0036	0.0151	103.7	157.0	119.6	53.3
0.550	0.0409	0.0144	-0.0030	0.0147	101.8	152.8	136.9	51.0
0.600	0.0383	0.0139	-0.0023	0.0141	99.3	146.2	151.5	46.9
0.650	0.0362	0.0135	-0.0017	0.0136	97.2	140.3	155.3	43.1
0.700	0.0343	0.0131	-0.0012	0.0131	95.4	135.1	151.5	39.7
0.750	0.0327	0.0127	-0.0009	0.0127	93.9	130.6	145.8	36.7
0.800	0.0308	0.0122	-0.0005	0.0122	92.2	125.5	137.8	33.3
0.850	0.0293	0.0117	-0.0001	0.0117	90.7	121.2	128.9	30.5
0.900	0.0278	0.0112	0.0001	0.0112	89.4	117.5	120.5	28.1
0.950	0.0266	0.0108	0.0003	0.0108	88.3	114.3	112.7	26.1
1.000	0.0251	0.0103	0.0005	0.0103	87.0	110.8	103.7	23.8

[a] BSS-C': the Sapporo-TZP+sp for Br and N with the 6-311+G(d,p) for C, H, F, O, and S. [b] Data are given for the interaction in question at the BCP. [c] w in $r = r_o + wa_o$ for TS [Me^{A}Br–N(Tf)-∗-^{B}BrMe]. [d] $c\nabla^2\rho_b(\boldsymbol{r}_c) = H_b(\boldsymbol{r}_c) - V_b(\boldsymbol{r}_c)/2$, where $c = \hbar^2/8m$. [e] $R = (x^2 + y^2)^{1/2}$, where $(x, y) = (H_b(\boldsymbol{r}_c) - V_b(\boldsymbol{r}_c)/2, H_b(\boldsymbol{r}_c))$. [f] $\theta = 90° - \tan^{-1}(y/x)$. [g] $\theta_p = 90° - \tan^{-1}(dy/dx)$. [h] $\kappa_p = |d^2y/dx^2|/[1 + (dy/dx)^2]^{3/2}$. [i] $\Delta\theta_p = \theta_p - \theta$.

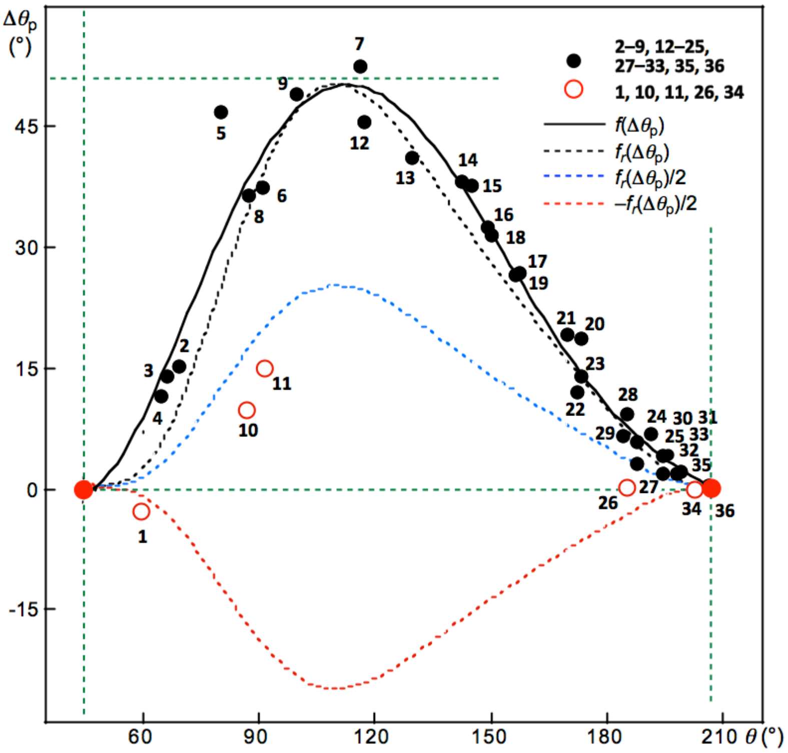

Figure S1. Plots of $\Delta\theta_p$ versus θ for the standard interactions of **1**–**36**, evaluated with MP2/BSS-B. Numbers are the same as those in Table S4. Red circles correspond to the θ period of 45.0° $\leq \theta \leq$ 206.6° and green horizontal lines are for $\Delta\theta_p$ = 0° and 50.0°.

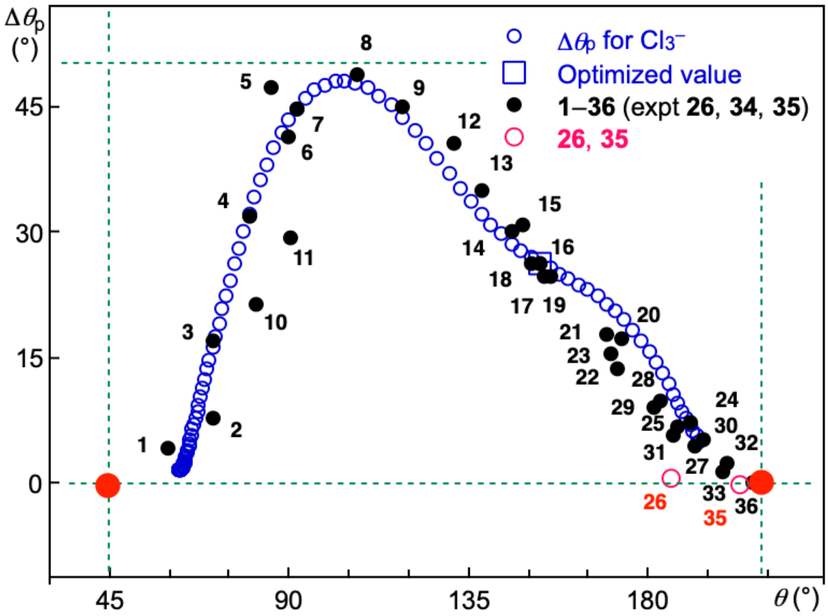

Figure S2. Plots of $\Delta\theta_p$ versus θ for the standard interactions, evaluated with MP2/BSS-A. Numbers are the same as those in Table S4, together with the wide range of the data of [Cl–Cl-∗-Cl]$^-$ (**16**). Red circles correspond to the θ period of 45.0° $\leq \theta \leq$ 206.6° and green horizontal lines are for $\Delta\theta_p$ = 0° and 50.0°.

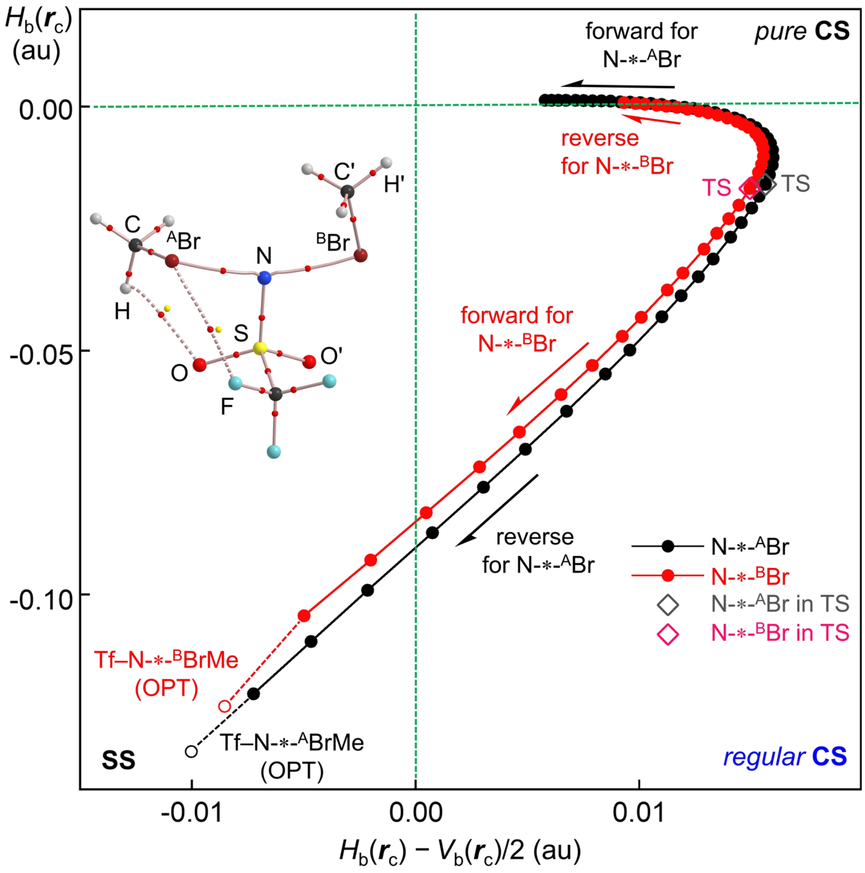

Figure S3. Plots of $H_b(\boldsymbol{r}_c)$ versus $H_b(\boldsymbol{r}_c) - V_b(\boldsymbol{r}_c)/2$ for wide range of the interaction distances around TS [Me^{A}Br-∗-N(Tf)-∗-^{B}BrMe], calculated MP2/BSS-C'. The perturbed structures are generated with IRC.

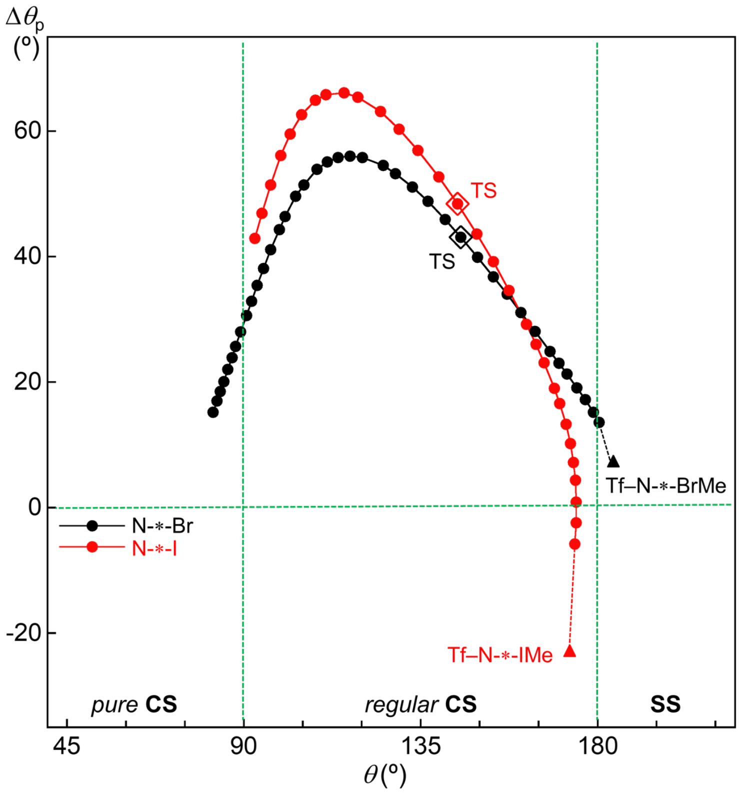

Figure S4. Plots of$_p\Delta\theta$ versus θ for the interaction distances around TS [MeI-∗-N(Tf)-∗-BrMe], calculated MP2/BSS-C'.

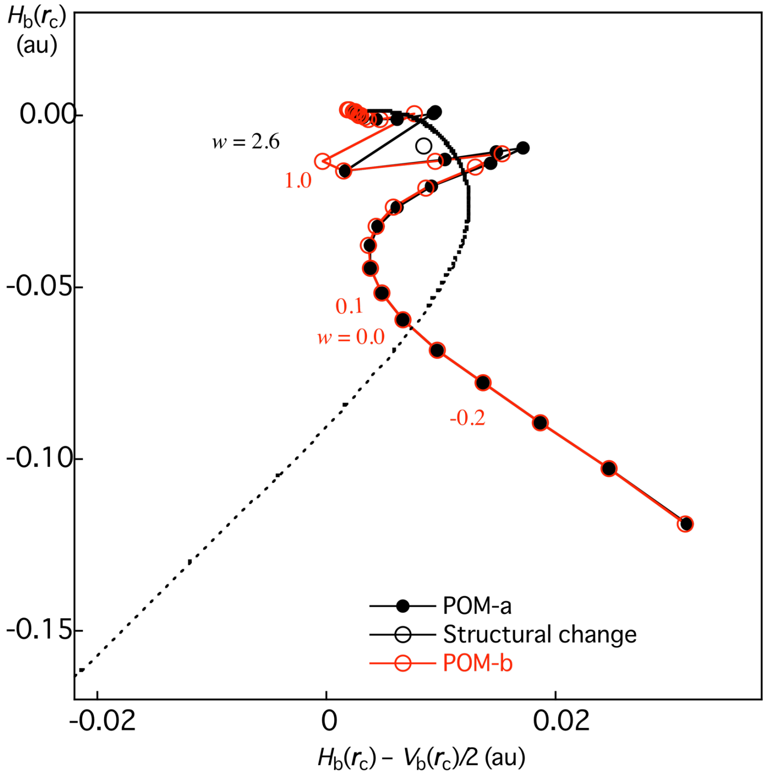

Figure S5. Plots of $H_b(\boldsymbol{r}_c)$ versus $H_b(\boldsymbol{r}_c) - V_b(\boldsymbol{r}_c)/2$ for wide range of the interaction distances for $[HS\text{-}*\text{-}TeH]^{2+}$, calculated MP2/BSS-C. Partially optimized with the S-∗-Te distances being variously fixed in POM-a, while the ∠TeSH and ∠STeH are additionally fixed as in the fully optimized structure in POM-b. The dotted lines correspond to the plot of the wide range of the data for $[Cl\text{–}Cl\text{-}*\text{-}Cl]^-$.

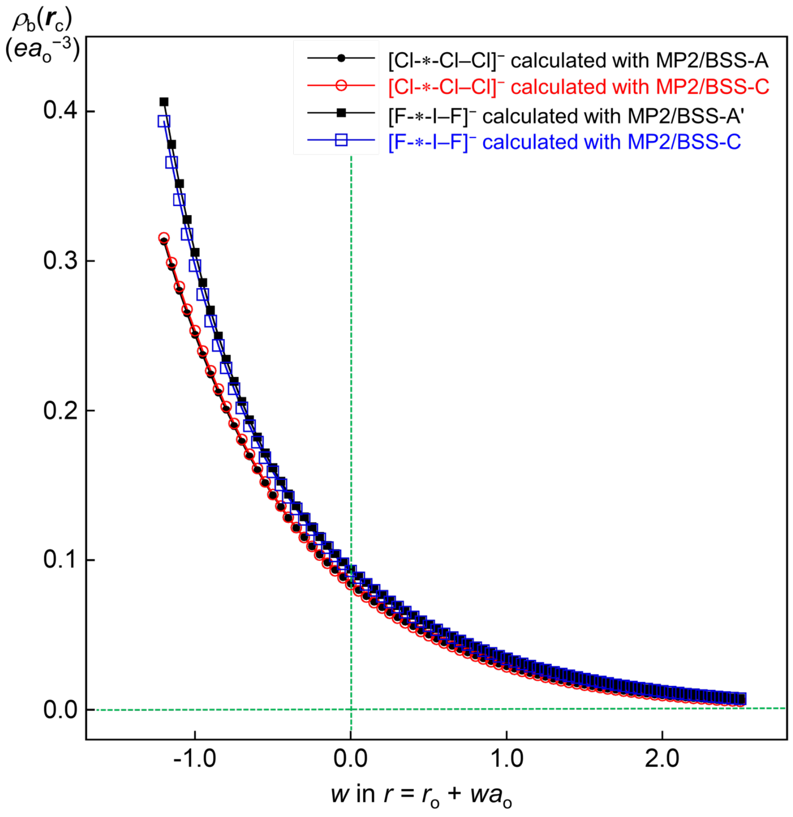

Figure S6. Plots of $\rho_b(\boldsymbol{r}_c)$ versus w in $r = r_o + wa_o$ for $[Cl\text{-}*\text{-}Cl\text{–}Cl]^-$ calculated with MP2/BSS-A and MP2/BSS-C, together with $[F\text{-}*\text{-}I\text{–}F]^-$ evaluated with MP2/BSS-A' and MP2/BSS-C.

Optimized structures

Gaussian 09 programs were employed for the calculations. The aug-cc-pVTZ and/or 6-311+G(3df,3pd) basis sets were applied to the atoms of the Groups 1st–4th elements in the calculations. They are called basis set system A (BSS-A) and basis set system B (BSS-B), respectively. The Sapporo-TZP basis sets with diffusion functions of the 1s1p type (abbreviated as S-TZPsp) were implemented from the Sapporo Basis Set Factory, which was called BSS-C. The S-TZPsp basis sets were applied to the atoms of the 5th period in addition to BSS-A and BSS-B, which were called BSS-A' and BSS-B', respectively. The Møller-Plesset second-order energy correlation (MP2) level was applied to the calculations. The optimized structures were confirmed by all real frequencies. The results were used to obtain the compliance constants (C_{ii}) and the coordinates corresponding to C_{ii} ($\boldsymbol{C}_i$). The optimizations were not corrected with the BSSE method.

MP2/BSS-C
Adduct MeSSMe (S, S)$_{Me}^0$
Symmetry C_2
Energy MP2 = –874.983811 au
Standard orientation

1	0	-0.267587	2.349295	0.570769
1	0	-1.982284	1.962620	0.846392
1	0	-0.729782	1.110627	1.769370
6	0	-0.971692	1.556499	0.807829
16	0	-0.971692	0.306598	-0.502094
16	0	0.971692	-0.306598	-0.502094
6	0	0.971692	-1.556499	0.807829
1	0	0.267587	-2.349295	0.570769
1	0	1.982284	-1.962620	0.846392
1	0	0.729782	-1.110627	1.769370

MP2/BSS-C
Adduct MeSSeMe (S, Se)$_{Me}^0$
Symmetry C_1
Energy MP2 = –2877.359698 au
Standard orientation

1	0	-2.107549	0.018056	-1.568819
1	0	-3.157876	0.705808	-0.308893
1	0	-1.712073	1.592099	-0.830373
6	0	-2.129238	0.603896	-0.654244
16	0	-1.227603	-0.251864	0.664820
34	0	0.756512	-0.429321	-0.200795
6	0	1.470473	1.306769	0.286626
1	0	1.440639	1.422010	1.365378
1	0	2.502286	1.327841	-0.060984
1	0	0.907417	2.096941	-0.200696

MP2/BSS-C
Adduct MeSTeMe (S, Te)$_{Me}^0$
Symmetry C_1
Energy MP2 = –7089.183479 au
Standard orientation

1	0	2.345222	-0.264748	1.569247
1	0	3.466399	0.568443	0.469335
1	0	2.042537	1.424620	1.093011
6	0	2.423398	0.461196	0.765074
16	0	1.528801	-0.121053	-0.707315
52	0	-0.675115	-0.357448	0.103183
6	0	-1.268346	1.670124	-0.181589
1	0	-1.138927	1.935199	-1.225890
1	0	-2.319635	1.749077	0.089894

1	0	-0.680756	2.323649	0.455011

MP2/BSS-C
Adduct MeSeSeMe (Se, Se)$_{Me}$^{0}
Symmetry C_2
Energy MP2 = –4879.737101 au
Standard orientation

1	0	1.564167	1.888030	0.833519
1	0	0.143484	2.904992	1.176616
1	0	0.356399	1.366816	2.040363
6	0	0.511786	1.883233	1.098152
34	0	-0.511786	1.029004	-0.312924
34	0	0.511786	-1.029004	-0.312924
6	0	-0.511786	-1.883233	1.098152
1	0	-1.564167	-1.888030	0.833519
1	0	-0.143484	-2.904992	1.176616
1	0	-0.356399	-1.366816	2.040363

MP2/BSS-C
Adduct MeSeTeMe (Se, Te)$_{Me}$^{0}
Symmetry C_1
Energy MP2 = –9091.561222 au
Standard orientation

1	0	1.895186	0.043502	2.010297
1	0	3.165162	0.792641	1.012616
1	0	1.642526	1.662225	1.305158
6	0	2.091690	0.685268	1.157747
34	0	1.391847	-0.139215	-0.460907
52	0	-1.000933	-0.335891	0.167129
6	0	-1.578482	1.625138	-0.443807
1	0	-1.340898	1.754266	-1.494615
1	0	-2.652704	1.718039	-0.294670
1	0	-1.062783	2.366510	0.157692

MP2/BSS-C
Adduct MeTeTeMe (Te, Te)$_{Me}$^{0}
Symmetry C_2
Energy MP2 = –13303.383975 au
Standard orientation

1	0	1.638293	2.045863	1.010741
1	0	0.274417	3.114055	1.414534
1	0	0.442191	1.546429	2.234726
6	0	0.593859	2.079886	1.302377
52	0	-0.593859	1.194183	-0.239890
52	0	0.593859	-1.194183	-0.239890
6	0	-0.593859	-2.079886	1.302377
1	0	-1.638293	-2.045863	1.010741
1	0	-0.274417	-3.114055	1.414534
1	0	-0.442191	-1.546429	2.234726

MP2/BSS-C
Adduct MeSSMe^{-} (S, S)$_{Me}$$^{-}$
Symmetry C_1
Energy MP2 = –874.982750 au
Standard orientation

1	0	-1.668837	0.469512	-1.645992
1	0	-2.703965	1.174750	-0.388767
1	0	-1.005649	1.660109	-0.525937
6	0	-1.695948	0.819096	-0.614266
16	0	-1.243821	-0.513677	0.531507
16	0	1.243803	-0.513679	-0.531509
6	0	1.695977	0.819075	0.614268
1	0	1.668894	0.469477	1.645991

1	0	2.703988	1.174728	0.388743
1	0	1.005677	1.660090	0.525970

MP2/BSS-C
Adduct MeSSeMe$^-$ (S, Se)$_{Me}^-$
Symmetry C_1
Energy MP2 = –2877.366796 au
Standard orientation

1	0	1.915446	-0.449651	1.679760
1	0	3.170379	0.583068	0.967540
1	0	1.543749	1.220916	1.255400
6	0	2.106371	0.336576	0.949760
16	0	1.625210	-0.201553	-0.717198
34	0	-1.021762	-0.427492	0.127972
6	0	-1.161770	1.478291	-0.251623
1	0	-1.029376	1.652827	-1.316540
1	0	-2.141694	1.840500	0.059700
1	0	-0.389561	2.022713	0.289447

MP2/BSS-C
Adduct MeSTeMe$^-$ (S, Te)$_{Me}^-$
Symmetry C_1
Energy MP2 = –7089.197507 au
Standard orientation

1	0	2.137392	-0.665387	1.640001
1	0	3.520212	0.238923	0.993913
1	0	1.998514	1.069171	1.351396
6	0	2.432910	0.142600	0.970901
16	0	1.880022	-0.196233	-0.728505
52	0	-0.890517	-0.304582	0.063608
6	0	-0.683096	1.823754	-0.150180
1	0	-0.446322	2.052787	-1.184808
1	0	-1.611041	2.313728	0.139885
1	0	0.128900	2.170647	0.483748

MP2/BSS-C
Adduct MeSeSeMe$^-$ (Se, Se)$_{Me}^-$
Symmetry C_1
Energy MP2 = –4879.750301 au
Standard orientation

1	0	-1.273364	0.784598	-1.958273
1	0	-2.621132	1.458651	-1.011962
1	0	-0.952084	1.967916	-0.685344
6	0	-1.582894	1.128766	-0.974504
34	0	-1.405246	-0.323052	0.315070
34	0	1.405247	-0.323051	-0.315071
6	0	1.582893	1.128764	0.974506
1	0	1.273338	0.784600	1.958269
1	0	2.621135	1.458632	1.011985
1	0	0.952103	1.967927	0.685336

MP2/BSS-C
Adduct MeSeTeMe$^-$ (Se, Te)$_{Me}^-$
Symmetry C_1
Energy MP2 = –9091.579790 au
Standard orientation

1	0	1.555095	-0.094398	2.113783
1	0	3.010300	0.747191	1.532230
1	0	1.420907	1.529079	1.427016
6	0	1.948263	0.578950	1.355743
34	0	1.698162	-0.206773	-0.413829
52	0	-1.260232	-0.296520	0.113238
6	0	-1.108342	1.751837	-0.522651

1	0	-0.717872	1.776376	-1.535463
1	0	-2.089668	2.221817	-0.486713
1	0	-0.423706	2.284515	0.132387

MP2/BSS-C
Adduct MeTeTeMe^{-} (Te, Te)$_{Me}$$^{-}$
Symmetry C_2
Energy MP2 = −13303.407267 au
Standard orientation

1	0	1.707779	1.667682	0.966495
1	0	0.569951	2.811739	1.723836
1	0	0.529882	1.085935	2.149447
6	0	0.699041	1.798949	1.346516
52	0	-0.699041	1.434679	-0.248440
52	0	0.699041	-1.434679	-0.248440
6	0	-0.699041	-1.798949	1.346516
1	0	-1.707779	-1.667682	0.966495
1	0	-0.569951	-2.811739	1.723836
1	0	-0.529882	-1.085935	2.149447

MP2/BSS-C
Adduct MeSSMe^{+} (S, S)$_{Me}$$^{+}$
Symmetry C_{2h}
Energy MP2 = −874.689162 au
Standard orientation

1	0	0.491854	2.342627	0.900720
1	0	2.031413	2.257132	0.000000
1	0	0.491854	2.342627	-0.900720
6	0	0.979442	1.978932	0.000000
16	0	0.979442	0.172208	0.000000
16	0	-0.979442	-0.172208	0.000000
6	0	-0.979442	-1.978932	0.000000
1	0	-0.491854	-2.342627	0.900720
1	0	-2.031413	-2.257132	0.000000
1	0	-0.491854	-2.342627	-0.900720

MP2/BSS-C
Adduct MeSSeMe^{+} (S, Se)$_{Me}$$^{+}$
Symmetry C_1
Energy MP2 = −2877.069143 au
Standard orientation

1	0	-2.388378	-0.939279	0.899957
1	0	-3.312072	0.295721	0.000167
1	0	-2.388449	-0.939058	-0.900000
6	0	-2.423284	-0.331225	0.000055
16	0	-1.031962	0.827688	0.000142
34	0	0.598433	-0.517580	-0.000086
6	0	2.001943	0.810681	0.000019
1	0	1.927473	1.411154	0.901497
1	0	2.926741	0.238069	-0.000085
1	0	1.927405	1.411369	-0.901311

MP2/BSS-C
Adduct MeSTeMe^{+} (S, Te)$_{Me}$$^{+}$
Symmetry C_1
Energy MP2 = −7088.900406 au
Standard orientation

1	0	-2.620060	-0.942328	0.899605
1	0	-3.582701	0.252997	0.000113
1	0	-2.620116	-0.942200	-0.899608
6	0	-2.669182	-0.336842	0.000043
16	0	-1.330776	0.901703	0.000088
52	0	0.549143	-0.431357	-0.000040

6	0	1.879111	1.210084	0.000031
1	0	1.706781	1.796747	0.898045
1	0	2.886756	0.801772	-0.000021
1	0	1.706735	1.796864	-0.897897

MP2/BSS-C
Adduct MeSeSeMe^{+} (Se, Se)$_{Me}$$^{+}$
Symmetry C_2
Energy MP2 = –4879.451442 au
Standard orientation

1	0	1.285951	2.159913	-0.900481
1	0	0.148515	3.201292	-0.000015
1	0	1.285925	2.159932	0.900506
6	0	0.687151	2.256845	0.000003
34	0	-0.687151	0.893387	-0.000001
34	0	0.687151	-0.893387	-0.000001
6	0	-0.687151	-2.256845	0.000003
1	0	-1.285951	-2.159913	-0.900481
1	0	-0.148515	-3.201292	-0.000015
1	0	-1.285925	-2.159932	0.900506

MP2/BSS-C
Adduct MeSeTeMe^{+} (Se, Te)$_{Me}$$^{+}$
Symmetry C_1
Energy MP2 = –9091.281344 au
Standard orientation

1	0	-2.213233	-1.541567	0.900221
1	0	-3.413198	-0.577050	0.000001
1	0	-2.213233	-1.541568	-0.900217
6	0	-2.398283	-0.965141	0.000002
34	0	-1.264373	0.617137	0.000000
52	0	0.900852	-0.472034	0.000000
6	0	1.979554	1.351338	0.000002
1	0	1.731024	1.909129	0.897898
1	0	3.034347	1.087840	-0.000006
1	0	1.731014	1.909136	-0.897887

MP2/BSS-C
Adduct MeTeTeMe^{+} (Te, Te)$_{Me}$$^{+}$
Symmetry C_2
Energy MP2 = –13303.110768 au
Standard orientation

1	0	-1.390419	2.335794	0.898202
1	0	-0.328001	3.449240	-0.000008
1	0	-1.390429	2.335783	-0.898193
6	0	-0.795691	2.468314	0.000000
52	0	0.795691	1.054184	0.000000
52	0	-0.795691	-1.054184	0.000000
6	0	0.795691	-2.468314	0.000000
1	0	1.390419	-2.335794	0.898202
1	0	0.328001	-3.449240	-0.000008
1	0	1.390429	-2.335783	-0.898193

MP2/BSS-C
Adduct MeSSMe^{2+} (S, S)$_{Me}$$^{2+}$
Symmetry C_{2h}
Energy MP2 = –874.159150 au
Standard orientation

1	0	0.062256	2.461248	0.000000
1	0	-1.523119	2.303675	-0.893404
1	0	-1.523119	2.303675	0.893404
6	0	-0.939760	2.033605	0.000000
16	0	-0.939760	0.251635	0.000000

16	0	0.939760	-0.251635	0.000000
6	0	0.939760	-2.033605	0.000000
1	0	-0.062256	-2.461248	0.000000
1	0	1.523119	-2.303675	-0.893404
1	0	1.523119	-2.303675	0.893404

MP2/BSS-C
Adduct MeSSeMe^{2+} (S, Se)$_{Me}$$^{2+}$
Symmetry C_1
Energy MP2 = –2876.550562 au
Standard orientation

1	0	-2.215857	-1.365935	-0.000024
1	0	-3.042605	-0.011348	0.893834
1	0	-3.042622	-0.011288	-0.893777
6	0	-2.476723	-0.309106	0.000014
16	0	-1.051055	0.771111	0.000037
34	0	0.612254	-0.491614	-0.000022
6	0	2.041138	0.786619	0.000004
1	0	1.665920	1.808619	0.000039
1	0	2.624465	0.545956	0.897025
1	0	2.624448	0.546011	-0.897043

MP2/BSS-C
Adduct MeSTeMe^{2+} (S, Te)$_{Me}$$^{2+}$
Symmetry C_1
Energy MP2 = –7088.402544 au
Standard orientation

1	0	2.447103	-1.344318	-0.000015
1	0	3.315113	-0.026633	-0.894636
1	0	3.315050	-0.026796	0.894909
6	0	2.744733	-0.299150	0.000091
16	0	1.344922	0.844227	0.000146
52	0	-0.561126	-0.417835	-0.000067
6	0	-1.909747	1.190054	0.000035
1	0	-1.372914	2.137735	0.000148
1	0	-2.527205	1.067270	-0.895152
1	0	-2.527277	1.067097	0.895148

MP2/BSS-C
Adduct MeSeSeMe^{2+} (Se, Se)$_{Me}$$^{2+}$
Symmetry C_2
Energy MP2 = –4878.943175 au
Standard orientation

1	0	0.418466	2.903020	-0.897156
1	0	0.418461	2.903017	0.897167
1	0	1.668272	1.935453	0.000008
6	0	0.650168	2.318654	0.000005
34	0	-0.650168	0.901833	-0.000001
34	0	0.650168	-0.901833	-0.000001
6	0	-0.650168	-2.318654	0.000005
1	0	-0.418466	-2.903020	-0.897156
1	0	-0.418461	-2.903017	0.897167
1	0	-1.668272	-1.935453	0.000008

MP2/BSS-C
Adduct MeSeTeMe^{2+} (Se, Te)$_{Me}$$^{2+}$
Symmetry C_1
Energy MP2 = –9090.793898 au
Standard orientation

1	0	-1.976764	-1.854755	0.000002
1	0	-3.100377	-0.751821	0.897277
1	0	-3.100375	-0.751820	-0.897274
6	0	-2.493757	-0.899520	0.000002

34	0	-1.266343	0.599405	0.000004
52	0	0.905026	-0.463253	-0.000003
6	0	2.058147	1.298368	0.000000
1	0	1.418385	2.178759	0.000001
1	0	2.683546	1.247976	0.895407
1	0	2.683547	1.247979	-0.895406

MP2/BSS-C
Adduct MeTeTeMe^{2+} (Te, Te)$_{Me}^{2+}$
Symmetry C_2
Energy MP2 = –13302.639724 au
Standard orientation

1	0	1.749146	2.086689	-0.000013
1	0	0.589282	3.139963	-0.895521
1	0	0.589298	3.139946	0.895537
6	0	0.761303	2.539640	0.000001
52	0	-0.761303	1.066995	0.000000
52	0	0.761303	-1.066995	0.000000
6	0	-0.761303	-2.539640	0.000001
1	0	-1.749146	-2.086689	-0.000013
1	0	-0.589282	-3.139963	-0.895521
1	0	-0.589298	-3.139946	0.895537

MP2/BSS-C
Adduct HSSH (S, S)$_H^0$
Symmetry C_2
Energy MP2 = –796.544145 au
Standard orientation

1	0	0.944275	1.214691	0.875539
16	0	0.000000	1.029492	-0.054721
16	0	0.000000	-1.029492	-0.054721
1	0	-0.944275	-1.214691	0.875539

MP2/BSS-C
Adduct HSSeH (S, Se)$_H^0$
Symmetry C_1
Energy MP2 = –2798.917826 au
Standard orientation

1	0	-0.915935	1.396420	0.027398
34	0	-0.715894	-0.044877	0.002769
16	0	1.472730	0.009180	-0.084742
1	0	1.692663	-0.017489	1.234342

MP2/BSS-C
Adduct HSTeH (S, Te)$_H^0$
Symmetry C_1
Energy MP2 = –7010.738463 au
Standard orientation

1	0	-0.771779	1.608706	0.006163
52	0	-0.577167	-0.033465	0.001931
16	0	1.798754	0.008005	-0.083995
1	0	2.004381	0.003398	1.237332

MP2/BSS-C
Adduct HSeSeH (Se, Se)$_H^0$
Symmetry C_2
Energy MP2 = –4801.292478 au
Standard orientation

1	0	1.027515	1.309697	0.989004
34	0	0.000000	1.158464	-0.029088
34	0	0.000000	-1.158464	-0.029088
1	0	-1.027515	-1.309697	0.989004

MP2/BSS-C
Adduct HSeTeH (Se, Te)$_H^0$
Symmetry C_1
Energy MP2 = –9013.112786 au
Standard orientation

1	0	-1.179172	1.609314	0.017513
52	0	-0.993795	-0.033262	0.001466
34	0	1.505339	0.003954	-0.043922
1	0	1.674999	-0.014107	1.399645

MP2/BSS-C
Adduct HTeTeH (Te, Te)$_H^0$
Symmetry C_2
Energy MP2 = –13224.930484 au
Standard orientation

1	0	1.167394	1.498634	1.137312
52	0	0.000000	1.343058	-0.021871
52	0	0.000000	-1.343058	-0.021871
1	0	-1.167394	-1.498634	1.137312

MP2/BSS-C
Adduct HSSH$^-$ (S, S)$_H^-$
Symmetry C_2
Energy MP2 = –796.563143 au
Standard orientation

1	0	0.976309	1.278277	0.853953
16	0	0.000000	1.363051	-0.053372
16	0	0.000000	-1.363051	-0.053372
1	0	-0.976309	-1.278277	0.853953

MP2/BSS-C
Adduct HSSeH$^-$ (S, Se)$_H^-$
Symmetry C_1
Energy MP2 = –2798.943761 au
Standard orientation

1	0	-0.868035	1.370897	0.329645
34	0	-0.919399	-0.039463	-0.009916
16	0	1.891715	0.021026	-0.074766
1	0	1.860145	-0.365578	1.203773

MP2/BSS-C
Adduct HSTeH$^-$ (S, Te)$_H^-$
Symmetry C_1
Energy MP2 = –7010.768328 au
Standard orientation

1	0	-0.583684	1.607046	0.160354
52	0	-0.714016	-0.029531	-0.002946
16	0	2.218377	0.008217	-0.078058
1	0	2.218471	-0.202896	1.241751

MP2/BSS-C
Adduct HSeSeH$^-$ (Se, Se)$_H^-$
Symmetry C_2
Energy MP2 = –4801.323774 au
Standard orientation

1	0	1.048177	1.391121	0.974198
34	0	0.000000	1.454579	-0.028653
34	0	0.000000	-1.454579	-0.028653
1	0	-1.048177	-1.391121	0.974198

MP2/BSS-C
Adduct HSeTeH$^-$ (Se, Te)$_H^-$
Symmetry C_1

Energy MP2 = –9013.147086 au
Standard orientation

1	0	-1.104794	1.598322	0.242115
52	0	-1.216325	-0.029572	-0.004694
34	0	1.839374	0.006414	-0.040629
1	0	1.814986	-0.278640	1.383334

MP2/BSS-C
Adduct HTeTeH^{-} (Te, Te)$_{H}^{-}$
Symmetry C_2
Energy MP2 = –13224.968340 au
Standard orientation

1	0	1.196463	1.518906	1.110451
52	0	0.000000	1.628880	-0.021355
52	0	0.000000	-1.628880	-0.021355
1	0	-1.196463	-1.518906	1.110451

MP2/BSS-C
Adduct HSSH^{2+} (S, S)$_{H}^{2+}$
Symmetry C_{2h}
Energy MP2 = –795.610238 au
Standard orientation

1	0	-1.375483	1.077287	0.000000
16	0	0.000000	0.977713	0.000000
16	0	0.000000	-0.977713	0.000000
1	0	1.375483	-1.077287	0.000000

MP2/BSS-C
Adduct HSSeH^{2+} (S, Se)$_{H}^{2+}$
Symmetry C_s
Energy MP2 = –2798.010448 au
Standard orientation

1	0	-1.486626	-0.730243	0.000000
34	0	0.002259	-0.687309	0.000000
16	0	0.002259	1.412143	0.000000
1	0	1.373684	1.504471	0.000000

MP2/BSS-C
Adduct HSTeH^{2+} (S, Te)$_{H}^{2+}$
Symmetry C_s
Energy MP2 = –7009.874997 au
Standard orientation

1	0	-1.670958	-0.538642	0.000000
52	0	0.004444	-0.558594	0.000000
16	0	0.004444	1.734323	0.000000
1	0	1.368732	1.836361	0.000000

MP2/BSS-C
Adduct HSeSeH^{2+} (Se, Se)$_{H}^{2+}$
Symmetry C_{2h}
Energy MP2 = –4800.409712 au
Standard orientation

1	0	-1.485440	1.156016	0.000000
34	0	0.000000	1.119953	0.000000
34	0	0.000000	-1.119953	0.000000
1	0	1.485440	-1.156016	0.000000

MP2/BSS-C
Adduct HSeTeH^{2+} (Se, Te)$_{H}^{2+}$
Symmetry C_s
Energy MP2 = –9012.269892 au
Standard orientation

1	0	-1.670870	-0.938907	0.000000

52	0	0.002192	-0.968080	0.000000
34	0	0.002192	1.463797	0.000000
1	0	1.482393	1.509936	0.000000

MP2/BSS-C
Adduct HTeTeH^{2+} (Te, Te)$_H^{2+}$
Symmetry C_{2h}
Energy MP2 = –13224.121425 au
Standard orientation

1	0	-1.670533	1.293309	0.000000
52	0	0.000000	1.321639	0.000000
52	0	0.000000	-1.321639	0.000000
1	0	1.670533	-1.293309	0.000000

MP2/BSS-A'
Adduct **41**: Me_2Te-F_2
Symmetry C_s
Energy MP2 = –6890.8677259 au
Standard orientation

52	0	0.494559	0.567278	0.000000
6	0	-0.903728	0.630202	1.554188
1	0	-0.369372	0.572678	2.498277
1	0	-1.537433	-0.244476	1.428124
1	0	-1.474123	1.553603	1.508554
6	0	-0.903728	0.630202	-1.554188
1	0	-0.369372	0.572678	-2.498277
1	0	-1.537433	-0.244476	-1.428124
1	0	-1.474123	1.553603	-1.508554
9	0	0.002562	-1.515493	0.000000
9	0	-0.903728	-3.020559	0.000000

MP2/BSS-A'
Adduct **46**: IF_2
Symmetry $D_{\infty h}$
Energy MP2 = –7117.6240045 au
Standard orientation

53	0	0.000000	0.000000	0.000000
9	0	0.000000	0.000000	2.071488
9	0	0.000000	0.000000	-2.071488

MP2/BSS-A'
Adduct **49**: Me_2FTeF
Symmetry C_2
Energy MP2 = –6891.0424097 au
Standard orientation

52	0	0.000000	0.000000	0.334629
6	0	-1.592153	-0.032869	-0.996418
6	0	1.592153	0.032869	-0.996418
1	0	2.454908	0.463280	-0.497490
1	0	1.307278	0.666632	-1.831236
1	0	1.789758	-0.987387	-1.307357
1	0	-2.454908	-0.463280	-0.497490
1	0	-1.307278	-0.666632	-1.831236
1	0	-1.789758	0.987387	-1.307357
9	0	0.000000	1.960970	0.101581
9	0	0.000000	-1.960970	0.101581

MP2/BSS-A'
Adduct **55**: Me_2TeF
Symmetry C_s
Energy MP2 = –6790.9944469 au
Standard orientation

52	0	-0.209850	0.299683	0.000000

6	0	-0.209850	-1.067202	1.553624
1	0	-0.038447	-0.532714	2.484324
1	0	0.576821	-1.793301	1.362507
1	0	-1.186683	-1.550258	1.578561
6	0	-0.209850	-1.067202	-1.553624
1	0	-0.038447	-0.532714	-2.484324
1	0	-1.186683	-1.550258	-1.578561
1	0	0.576821	-1.793301	-1.362507
9	0	1.636335	0.552829	0.000000

MP2/BSS-A
Adduct **60**: CH_3F
Symmetry C_{3v}
Energy MP2 = –139.5415326 au
Standard orientation

6	0	0.000000	0.000000	-0.636159
1	0	1.029721	0.000000	-0.984433
1	0	-0.514861	0.891765	-0.984433
1	0	-0.514861	-0.891765	-0.984433
9	0	0.000000	0.000000	0.752250
